# Long and short photoperiod buds in hybrid aspen share structural development and expression patterns of marker genes

**DOI:** 10.1093/jxb/erv380

**Published:** 2015-08-05

**Authors:** Päivi L.H. Rinne, Laju K. Paul, Jorma Vahala, Raili Ruonala, Jaakko Kangasjärvi, Christiaan van der Schoot

**Affiliations:** ^1^Department of Plant Sciences, Norwegian University of Life Sciences, N-1432 Ås, Norway; ^2^Division of Plant Biology, Department of Biosciences, University of Helsinki, FI-00014 Helsinki, Finland; ^3^College of Science, King Saud University, Riyadh 11451, Saudi Arabia

**Keywords:** Apical dominance, axillary bud, branching, BRC1-like, CENL1, dormancy, MAX1-like, PINL1-like, terminal bud.

## Abstract

Tree architecture develops over time through the collective activity of apical and axillary meristems. Although the capacity of both meristems to form buds is crucial for perennial life, a comparative analysis is lacking. As shown here for hybrid aspen, axillary meristems engage in an elaborate process of axillary bud (AXB) formation, while apical dominance prevents outgrowth of branches. Development ceased when AXBs had formed an embryonic shoot (ES) with a predictable number of embryonic leaves at the bud maturation point (BMP). Under short days, terminal buds (TBs) formed an ES similar to that of AXBs, and both the TB and young AXBs above the BMP established dormancy. Quantitative PCR and *in situ* hybridizations showed that this shared ability and structural similarity was reflected at the molecular level. TBs and AXBs similarly regulated expression of meristem-specific and bud/branching-related genes, including *CENTRORADIALIS-LIKE1* (*CENL1*), *BRANCHED1* (*BRC1*), *BRC2*, and the strigolactone biosynthesis gene *MORE AXILLARY BRANCHES1* (*MAX1*). Below the BMP, AXBs maintained high *CENL1* expression at the rib meristem, suggesting that it serves to maintain poise for growth. In support of this, decapitation initiated outgrowth of *CENL1*-expressing AXBs, but not of dormant AXBs that had switched *CENL1* off. This singles out *CENL1* as a rib meristem marker for para-dormancy. *BRC1* and *MAX1* genes, which may counterbalance *CENL1*, were down-regulated in decapitation-activated AXBs. The results showed that removal of apical dominance shifted AXB gene expression toward that of apices, while developing TBs adopted the expression pattern of para-dormant AXBs. Bud development thus follows a shared developmental pattern at terminal and axillary positions, despite being triggered by short days and apical dominance, respectively.

## Highlight

Short photoperiod and apical dominance trigger a shared developmental bud programme at terminal and axillary positions, while the capacity to establish photoperiod-induced dormancy is lost in maturing para-dormant axillary buds.

## Introduction

The distinctive architecture of a tree is derived from the collective activity of shoot meristems, seated at the apex and formed laterally in the axils of leaves. While the shoot apical meristem (SAM) extends the main axis, branches arise from axillary meristems (AXMs). Each species has a genetic ground plan, referred to as its ‘architectural model’ (Hallé *et al.*, 1978; Tomlinson, 1983; Millet *et al.*, 1999). However, the intricate and detailed form of the crown that emerges over time is the result of internal developmental competition between branches, and interaction with the external environment (Tomlinson, 1983; Barthélémy and Caraglio, 2007; Pfennig *et al.*, 2010; Donnelly *et al.*, 2012). The overall plasticity of development is vividly illustrated in the practice of bonsai, in which seedlings (‘sai’) of potentially huge trees are forced to grow in a miniaturized form in a ‘bon’, a tray-like pot. For temperate perennials, seasonal change is a major force that constrains and modulates the architectural process. This is particularly evident in deciduous woody perennials, where new shoots arise in spring from buds that overwintered on existing structures, which themselves were affected by past weather conditions.

The SAM, the basic organization of which is shared by all angiosperms (Sussex, 1989; Sachs, 1991; van der Schoot and Rinne, 1999; Jürgens, 2003), is the ultimate origin of the shoot system and its architectural layout (Sussex, 1989; Sachs, 1991). Nonetheless, different branching strategies have evolved. For example, in *Arabidopsis*, the formation of AXMs is delayed. Although in this species the identity of a leaf axil is initially secured by expression of *LATERAL SUPPRESSOR* (Greb *et al.*, 2003) and *REGULATOR OF AXILLARY MERISTEM1* (Keller *et al.*, 2006), the meristem identity gene *SHOOT MERISTEMLESS* (*STM*) is expressed first in the axils of older rosette leaves (Grbic and Bleecker, 2000; Long and Barton, 2000; Greb *et al.*, 2003). In contrast, in many angiosperms, including tree species, AXMs emerge in initial continuity with the SAM to give rise to axillary buds (AXBs) (Garrison, 1955; Esau, 1977).

The architectural possibilities of a tree crown are phyllotactically pre-determined, but realized only through differential activation of AXBs and outgrowth into branches. Some AXBs may remain inhibited for decades (Rinne *et al.*, 1993; Meier *et al.*, 2012). Adventitious branches, which do not follow the phyllotactic pattern, develop only under special circumstances, for example when all buds are removed (Rinne *et al.*, 1987). Despite their significance, AXB formation and branching in woody perennials are scantily investigated. Woody perennials employ two branching strategies based on the timing of AXB outgrowth. In sylleptic branching, AXBs give rise to branches in the same season, while in ‘proleptic’ (Hallé *et al.*, 1978) or ‘delayed’ (Barthélémy and Caraglio, 2007) branching the AXBs can only grow out after they have passed through a dormancy period (Hallé *et al.*, 1978). Syllepsis is common in tropical species (Hallé *et al.*, 1978; Cline and Dong-Il, 2002), but it also occurs in some temperate species (Ceulemans *et al.*, 1990; Wu and Hinckley, 2001) in which prolepsis prevails. Syllepsis is strongly modulated by environmental factors (Wu and Settler, 1998; Ceulemans *et al.*, 1990; Wu and Hinckley, 2001), thereby contributing to crown plasticity, while the relatively stable proleptic branching style is thought to be under strong apical dominance (Cline, 1997).

Conventionally, apical dominance denotes the phenomenon whereby AXBs are held captive in an inactive state by a proliferating apex. In this view, the inactive state, referred to as para-dormancy (Lang *et al.*, 1987), is enforced by auxin that is produced by the apex (Thimann and Skoog, 1934; Phillips, 1975; Cline, 1991, 1997). Indeed, simply removing the sources of auxin by decapitation abolishes apical dominance and removes AXB inhibition (Rinne *et al.*, 1993; Cline, 1997). In recent years, significant progress has been made with herbaceous species in uncovering the mechanisms that regulate branching, and the emerging concepts may serve as a heuristic paradigm for branching in proleptic hybrid aspen. In annuals, branching involves genetic controls, auxin transport, as well as long-distance signalling (Wang and Li, 2006; Hamiaux *et al.*, 2012; Brewer *et al.*, 2013; Wang *et al.*, 2014a; Wang *et al.*, 2014b). In one model, branching requires the production and export of auxin from the activated AXB to the polar auxin transport stream (PATS) in the xylem parenchyma of the stem (Li and Bangerth, 1999). In a situation in which the apex monopolizes the PATS by ‘saturating’ its transport capacity, this would be difficult to achieve (Domagalska and Leyser, 2011). A prerequisite for branching is the production and positioning of PINFORMED1 (PIN1) auxin efflux carriers in the plasma membrane of cells between the bud and the stem (Balla *et al.*, 2011; Domagalska and Leyser, 2011). Simply decapitating the plant will remove the dominant auxin source, and allow some activated AXBs to compete for access to the stem PATS. On the other hand, vigorously proliferating apices are not incompatible with branching, suggesting that important additional mechanisms are involved (Morris *et al.*, 2005; Dun *et al.*, 2006; Kitazawa *et al.*, 2008). A downstream target of apically produced auxin is strigolactone, the biosynthesis of which may require *MORE AXILLARY BRANCHES1* (*MAX1*) (Booker *et al.*, 2005). Auxin and phosphate starvation may promote MAX1-mediated strigolactone biosynthesis in roots (Gomez-Roldan *et al.*, 2008; Umehara *et al.*, 2008). From there, strigolactone is translocated to the AXBs, where it inhibits branching (Ruyter-Spira *et al.*, 2013). The inhibitory effect of strigolactone is counteracted by the promoting effect of root-produced cytokinins (Gomez-Roldan *et al.*, 2008; Ferguson and Beveridge, 2009). As strigolactone diminishes PIN1-mediated PATS, and enhances competition between activated AXBs, this process remains a nexus of the mechanisms that control branching (Domagalska and Leyser, 2011).

The genes that regulate branching in annuals may also play a role in deciduous trees because the basic control mechanisms are conserved among angiosperms (Wang and Li, 2006). On the other hand, emergent layers of regulation must be in place to account for the existence of juvenile and adult tree stages, and the unique presence of a seasonal dormancy cycle. This complexity was recognized by Brown *et al.* (1967) who referred to the mechanism that supervises the overall shape and form of the crown via various branching processes as ‘apical control’. Even though apical dominance might not be sufficient to explain branching in woody shoot systems (Wareing, 1970), it is important in preventing branching in current-year shoots of older trees (Cline, 1997). Nonetheless, even in current-year shoots, the situation is more complex than in annuals due to their overwintering capacity.

In hybrid aspen, vegetative current-year AXBs can be in more than one state. They can be quiescent, an inactive state which *sensu lato* includes both para-dormancy and eco-dormancy, as well as dormant, a state in which the AXM is arrested by an intrinsic mechanism that is triggered by short days. Para-dormancy, due to apical dominance, can be abolished by decapitation, but dormancy is insensitive to decapitation and requires prolonged chilling. Although these different states can be established and defined experimentally (Lang *et al.*, 1987; van der Schoot and Rinne, 2011), the similarities and differences at the molecular level are not well understood. In addition, AXBs and terminal buds (TBs) are formed at different phases of the seasonal cycle. AXBs develop under long days from AXMs that arise as daughter meristems from the SAM, while TBs develop under short days from a transitioning SAM (Wareing, 1956; Romberger, 1963; Rinne and van der Schoot, 1998; Rohde *et al.*, 2002).

Dormancy research has mostly focused on TBs, which are initiated under short days after the complete down-regulation of *FLOWERING LOCUS T* (*FT*) in the leaves (Böhlenius *et al.*, 2006; Hsu *et al.*, 2006, 2011; Ruonala *et al.*, 2008), and the early and gradual closing of plasmodesmata (PD) in the SAM (Rinne and van der Schoot, 1998; Ruonala *et al.*, 2008). Extensive shifts were observed in the transcriptome of developing TBs, particularly in relation to ethylene and abscisic acid (ABA) signalling (Ruttink *et al.*, 2007), while expression of dormancy-associated MADS box genes has been reported for some species (Horvath *et al.*, 2010; Jiménez *et al.*, 2010). Although AXBs can also establish dormancy under short days, it is uncertain if all AXBs have this capacity and which molecular mechanisms are involved.

An intriguing problem is how the AXBs and TBs safeguard the integrity of their SAM during dormancy. In annuals, meristem maintenance and functioning require the co-ordinated action of *WUSCHEL* (*WUS*) and *CLAVATA3* (*CLV3*), which balances cell proliferation in the SAM, while the knotted-like homeobox (*KNOX*) gene knotted1 (*KN1*) prevents premature differentiation (Jackson *et al.*, 1994; Laux *et al.*, 1996; Schoof *et al.*, 2000). WUS travels though PD to overlying stem cells to regulate *CLV3* (Daum *et al.*, 2014). In hybrid aspen, such movement must be absent in dormancy as PD are blocked (Rinne and van der Schoot, 1998; Rinne *et al.*, 2001; Ruonala *et al.*, 2008). In *Arabidopsis*, experimentally induced blockage of PD distorts or terminates the SAM (Daum *et al.*, 2014). In woody perennials, where the cellular uncoupling mechanism is part of their natural survival strategy, this does not happen. It remained unknown how *WUS*/*CLV*/*KN1* are regulated during dormancy establishment in both TB and AXBs.

A gene that might be important in AXB activation is *CENTRORADIALIS-LIKE1* (*CENL1*), the *Populus* orthologue of the *Arabidopsis* meristem-identity gene *TERMINAL FLOWER1* (*TFL1*). *CENL1* is expressed and up-regulated in the rib meristem of the apex during TB development, but switched off during dormancy (Ruonala *et al.*, 2008). *CENL1* as well as the tomato orthologue *SELF-PRUNING* (*SP*) are also expressed in para-dormant AXBs of *Populus* and tomato, respectively (Pnueli *et al.*, 1998; Mohamed *et al.*, 2010; Lifschitz *et al.*, 2014). The fact that *TFL1*/*SP*/*CENL1* are expressed in vegetative AXBs, and that in *Arabidopsis TFL1* influences branching (Ratcliffe *et al.*, 1998), suggests that they promote branching. If so, inhibitory forces must keep AXBs in check, as in hybrid aspen branching does not occur in the first year. In non-woody species, branch inhibition genes include members of the *TCP* family (*TEOSINTE BRANCHED1*, *CYCLOIDEA*, and *PCF*), which encode transcription factors that operate exclusively within AXBs (Hubbard *et al.*, 2002). In *Arabidopsis*, two orthologues have been identified, *BRANCHED1* (*BRC1*) and *BRC2* (Aguilar-Martínez *et al.*, 2007; Finlayson, 2007; Niwa *et al.*, 2013), but in trees these genes have not been studied.

The genus *Populus* displays considerable variation in branching styles among species and genotypes within species (Wu and Hinckley, 2001). The present work addresses the structural development of AXBs and TBs in first-year saplings of hybrid aspen (*Populus tremula x P. tremuloides*), clone T89. This clone has a strictly proleptic branching style, a sign of strong apical dominance (Cline, 1997), and therefore is ideally suited to investigate the endogenous mechanisms that govern both AXB development and its outgrowth to a branch. The commonalities and differences between para-dormant and dormant states, as well as branch initiation, were investigated through molecular analyses. The data showed that TBs and AXBs both produce an identically regulated dwarfed shoot system, despite the fact that their development is under control of distinct triggers, apical dominance and short photoperiod, and in different seasons. Both require up-regulation of *CENL1*, but whereas in para-dormancy *CENL1* expression is maintained, in dormancy it is completely down-regulated. The results suggest a working model in which two distinct signalling pathways, short days and apical dominance, converge on a shared developmental programme for bud formation. So long as buds are in the early phase of development they remain susceptible to short days and capable of establishing dormancy.

## Materials and methods

### Plant material and designs for experiments

Hybrid aspen (*Populus tremula*×*P. tremuloides*) clone T89, and lines overexpressing oat (*Avena sativa*) phytochrome A (*PHYA*) (line 22; Ruonala *et al.*, 2008) were micro-propagated *in vitro*, planted in soil, and grown in a greenhouse under long days (18h light) at ~18 °C and 75–80% relative humidity (RH), and watered twice a day. Natural light was supplemented to 200 μmol m^–2^ s^–1^ at 400–750nm (Osram). After 6 weeks, when the plants were 70–80cm tall and elongation and leaf production rates were constant, the plants were subdivided into three groups. Group one was kept in long days as a control. Group two was moved to short days (10h) for minimally 5 weeks to induce dormancy. Group three was decapitated just above a node at pre-determined distances from the apex (see below) to remove apical dominance. Following decapitation, the kinetics of AXB activation were measured for a number of consecutive days, initially with a digital micrometer (World Precision Instruments, USA) and subsequently with a ruler. Dormancy establishment after 7 weeks of short days was monitored in TBs and AXBs at different stem positions, using bud-internode cuttings under growth-promoting conditions [18h of long day, photosynthetic photon flux density (PPFD) 200 μmol m^–2^ s^–1^, 18 ^○^C, and 85% RH] at weekly intervals for 3 weeks (Rinne *et al.*, 2011). This bud-internode system was also used to assess whether a xylem-fed synthetic strigolactone analogue (GR24) (Chiralix BV, The Netherlands) at concentrations of 0.5–5 μM inhibits AXB burst in hybrid aspen (*n*=3).

### AXB and embryonic shoot development

AXB enlargement and embryonic shoot (ES) ontogeny were investigated in eight proliferating plants. From each plant the 30 uppermost AXBs were collected and their size measured under a dissection microscope. Subsequently, the AXBs were fixed in 70% alcohol, and prepared under the dissection microscope to record number, type, and position of scales, leaves, stipules, and primordia. Representative examples were documented in photographs. The information obtained was used to determine three nodal positions for stem decapitation: (i) an early stage of AXB development; (ii) a more advanced stage of development; and (iii) a mature AXB stage.

### AXB anatomy

For light microscopy, AXBs were fixed overnight at 4 °C in 2% (v/v) glutaraldehyde and 3% (v/v) paraformaldehyde in 100mM phosphate citrate buffer (Rinne *et al.*, 2001). Briefly, samples were infiltrated gradually with LR White Resin (LRW) of increasing concentration (30–70%), and kept for 4 d in 100% LRW. Polymerization was done at 55 °C for 24h. Median longitudinal sections, 1–3 μm thick, were stained with 1% aqueous Toluidine blue.

### *In situ* hybridization of *CENL1*

The expression domain of *CENL1* in apices and AXBs of wild-type hybrid aspen (clone T89) and transgenic lines ectopically overexpressing the oat *PHYA* gene (Ruonala *et al.*, 2008) was visualized by a standard *in situ* hybridization technique, using digoxigenin and a Dig RNA Labeling kit (Roche) with modifications (Ruonala *et al.*, 2008). Antisense and sense RNA probes were prepared from the *CENL1* gene (Potri.004G203900, AY383600). To clone partial cDNA of Pt*CENL1*, RNA was isolated from apices after plants were exposed to short days for 2 weeks, a time point at which *CENL1* is up-regulated (Ruonala *et al.*, 2008). cDNA was amplified using Phusion (F-530, Thermo Fisher Scientific). Forward and reverse primers for amplification were 5′-TCATGGCAAAGATGTCAGAGC and 5′-CTTTGGGCATTGAAGAAGACA, respectively. The PCR product was cloned into the *Eco*RV site (blunt-end cloning) of the pZErO-2 vector, which has T7 and SP6 sites flanking the multiple cloning site. The colour reaction to visualize the hybridized probe was carried out at room temperature for up to 20h. Sections were examined and photographed with a Zeiss Axioplan2, and an Olympus AX70 equipped with an Olympus DP70 digital camera.

### RNA extraction and quantitative real-time PCR analysis

The apex and every second AXB between nodes 2 and 30 were collected from long-day plants. In parallel, apices and developing (growing) AXBs at nodal positions 2–14 were similarly collected from plants at short day week 2, 3, and 5. Two types of decapitation experiments were carried out. In the first experiment, the five AXBs immediately under the cut were analysed at 8 d and 14 d (not shown) post-decapitation. Based on these data, a second experiment was carried out in which the proximal AXB was collected after 1, 2, 3, 5, and 7 d. RNA was extracted from six plants and divided into two biological replicates, each containing material from three plants. Some of the investigated genes, such as *PINL1*, *CLV1*, and *WUS*, are potentially under diurnal regulation (http://diurnal.mocklerlab.org/). To make sure that the measured changes in gene expression were due to decapitation, and not to circadian variation, sampling was carried out at the same time of the day (Ruonala *et al.*, 2008; Rinne *et al.*, 2011). Specifically, all buds were harvested within the last hour of the light period under short days, and during the same hour in experiments with intact and decapitated long-day plants.

RNA was extracted from 0.2g of frozen tissue, and ground in a mortar with 750 μl of extraction buffer (Qiagen RTL buffer, containing 1% PVP-40). After addition of a 0.4 volume of KoAC at pH 6.5 and further grinding, the solution was transferred to a 2ml tube, incubated on ice for 15min, and centrifuged at 12 000rpm at 4 °C for 15min. The supernatant was transferred to a 1.5ml tube, and a 0.5 volume of 100% ethanol was added. The mix was transferred to two RNeasy-spin columns and further processed in accordance with instructions of the Qiagen Plant RNA isolation kit. RNA was DNase (Ambion) treated, cleaned using the total RNA purification system ‘Purelink RNA mini kit’ (Invitrogen), and reverse transcribed using SuperScriptIII reverse transcriptase (Invitrogen). Quantitative real-time PCR (qPCR) analyses were performed with the ABA Prism 7500Fast sequence detection system using SYBR Green PCR master mix (Applied Biosystems). Transcript levels were normalized using an actin gene. Gene-specific primer sequences for the analyses were designed using Primer3 (http://frodo.wi.mit.edu/primer3) (Supplementary Table S1 available at *JXB* online).

### Bioinformatics

Phylogenetic analyses of *Arabidopsis thaliana MAX1*, *BRC1*, and *BRC2* were carried out to identify orthologous proteins in perennial species with protein–protein BLAST searches in GenBank and the *Populus trichocarpa* genome v2.0 (Tuskan *et al.*, 2006) databases (http://www.ncbi.nlm.nih.gov/BLAST; http://www.phytozome.net). ClustalW (http://www.ebi.ac.uk/Tools/msa/clustalw2) was used to perform multiple sequence alignments. A phylogenetic tree was created using the MEGA5 program (www.megasoftware.net) with the Neighbor–Joining method. Bootstrap support values are based on 1000 replicates.

### Accession numbers

The *P. trichocarpa* gene model identifiers (Tuskan *et al.*, 2006) and/or sequence accessions used for qPCR analysis are listed in Supplementary Table S1 at *JXB* online.

## Results

### Young AXBs contain a morphogenetically active AXM

**Fig. 1.**
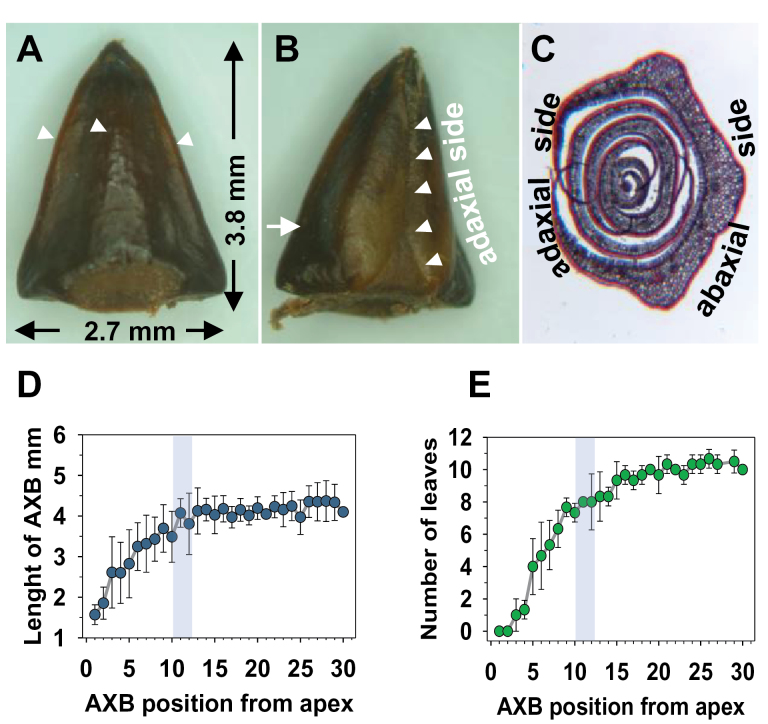
Axillary bud development and structural details. (A) Abaxial face of the outer scale of a mature axillary bud (AXB), armoured with three ridges (arrowheads). (B) Adaxial side of the outer scale of a mature AXB. Overlapping scale edge (arrowheads). The arrow indicates the position of the cross-section through the AXB, as in C. (C) Cross-section of an AXB; the position of the axillary meristem is below the section plane. All scales are simple and lack conductive tissue. (D) AXB enlargement and (E) number of embryonic leaves the AXBs contain at different stages of their development. Shadowed areas indicate the approximate bud maturation point (BMP). Values represent means ±SD (*n*=8 plants). Toluidine blue staining (C).

To map the developmental context in which para-dormancy, dormancy, and branching occur in hybrid aspen, the structural development and maturation of AXBs was investigated and compared with that of short-day-induced TBs. Morphometric analyses showed that the size of AXBs increased in the basipetal direction along the juvenile stem, but only up to a certain point, which was dubbed the ‘bud maturation point’ (BMP) (Fig. 1A–D). The increase in AXB size could not be due to a gradual activation of para-dormant AXBs because the hybrid aspen clone T89 delays branching to the next growing season. Surgical investigations of ~200 buds showed that the increase was due to the internal development of a dwarfed ES system (Romberger, 1963). Once the BMP was reached, the enclosed AXMs had commonly produced five scales and 10 ‘embryonic leaves’ (Fig. 1E).

**Fig. 2.**
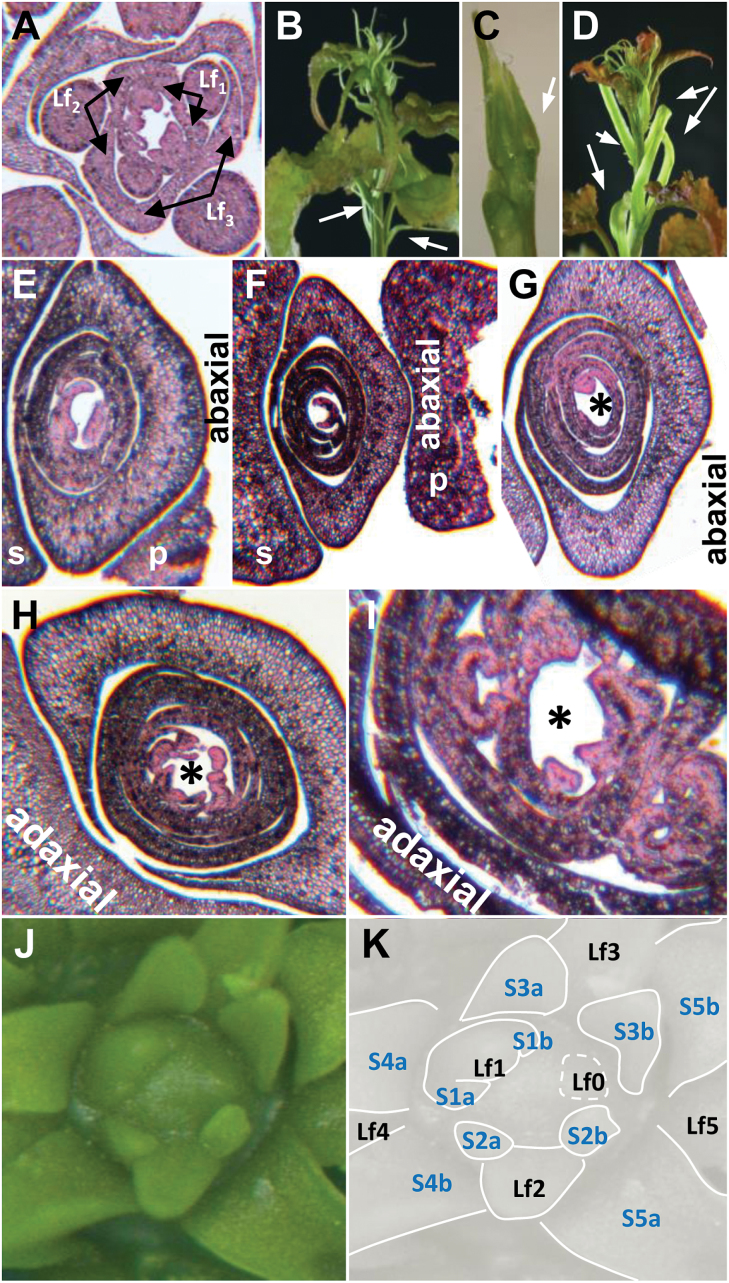
Bud development at apical and axillary positions. (A–D) Short photoperiod-induced development of a terminal bud (TB) at the shoot apex. (E–K) Default axillary bud (AXB) development under long photoperiod. (A) Stipules, paired leaf base extensions, are initiated early during leaf development. Arrows indicate stipules of three leaf primordia (Lf1–Lf3). Three younger leaf primordia are visible in the centre. The meristem is below the section plane. (B) Stipules are paired thread-like structures that form under long days (arrows), but which under short days metamorphose into scales while the leaf lamina fails to develop. (C) An emerging TB, after a 2-week exposure to short days. Overarching leaves were surgically removed (note brownish colour on the developing scale, arrow). (D) Reversion of the apex to a regular growth pattern under long days, after a restricted 2–3 week short day exposure. Note the reverted scale-like stipules (arrows). (E) Immature AXBs with the first three scales, (F) all five scales, and subsequently one (G), three (H) and seven (I) embryonic leaves. The asterisk in (G–I) points to the axillary meristem position, just below the section plane. (J) Immature fixed AXB, opened under the microscope (digitally coloured, Photoshop). (K) Diagram of (J), depicting the arrangement of embryonic leaves. The youngest leaf buttress is Lf0. Each embryonic leaf has two stipules (e.g. S1a and S1b, stipules of Lf1). (A, E–I) Transverse sections, toluidine blue staining. s, stem; p, petiole.

**Fig. 3.**
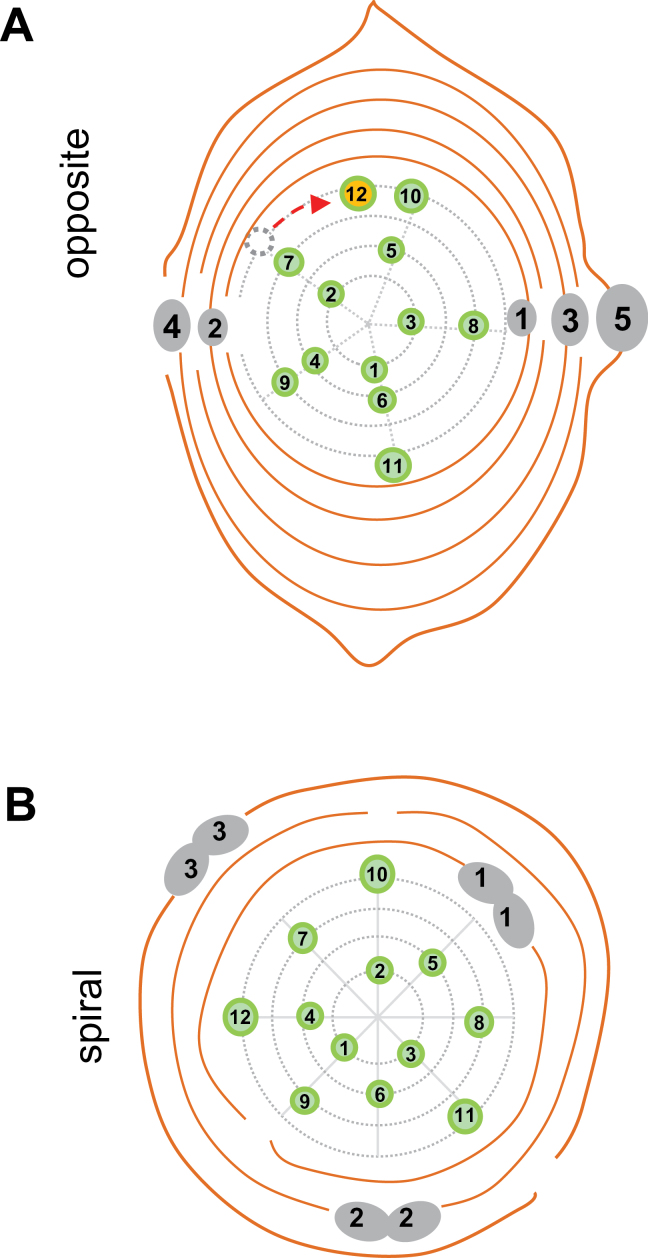
Schematic diagrams depicting primordia development and patterning in the apex (terminal bud) and axillary bud. (A) The first five primordia in axillary buds develop with opposite (decussate) arrangement (180°) and become scales. Number 5 is the abaxial outer scale. Subsequent 12 primordia develop into dwarfed embryonic leaves in a spiral pattern 2/5 (144°). The first embryonic leaf has a 90° divergence angle due to space constrains (red stippled arrow). (B) In the shoot apex the stipules of the first three (to five) primordia develop into scales (dual numbered brown lines) and the subsequent 12 primordia develop into dwarfed embryonic leaves. All primordia arise with 3/8 spiral pattern (135°) similar to proliferating apex.

The scales of AXBs were distinct from those of TBs induced by short days. Whereas the paired scales of TBs developed from the stipules of transformed leaves (Fig. 2A–D), the scales of AXBs were ‘perfect scales’ that arose directly from the AXM without metamorphosis, and prior to leaf production. The perfect scale primordia were produced in an alternate pattern, with a 180° divergence angle, which resulted in a flattened overall shape (Figs 2E–K, 3A), whereas scales of TBs were positioned in a radial pattern (Fig. 3B). In AXBs, from leaf primordium six and onward, there was an abrupt change in fate, and primordia developed into miniaturized, embryonic leaves that arose in a spiral pattern. The ES of AXBs was highly similar to that of TBs, although in AXBs the divergence angle was larger, 2/5 (144°) compared with 3/8 (137°) in TBs (Fig. 3). The angle between the first two embryonic leaves in AXBs was deviant, enforced by the flattened shape of the AXB (Fig. 3A). Below the BMP, the AXMs ceased primordia production, although the youngest primordia would still become more pronounced and leaf like (Fig. 1D, E), tightly packing the bud space. It took ~4 weeks for a newly initiated AXB to reach the BMP, and during this period the proliferating SAM of the main stem had produced ~10 younger phytomers. The virtual absence of cell elongation in the rib meristem of the ES ensured that the AXB remained closed even after the BMP was reached. Briefly, the present data show that up to the BMP an AXB contains a growing and developing ES, and that AXBs become morphogenetically inactive only below this point.

### AXBs are poised to burst around the BMP

**Fig. 4.**
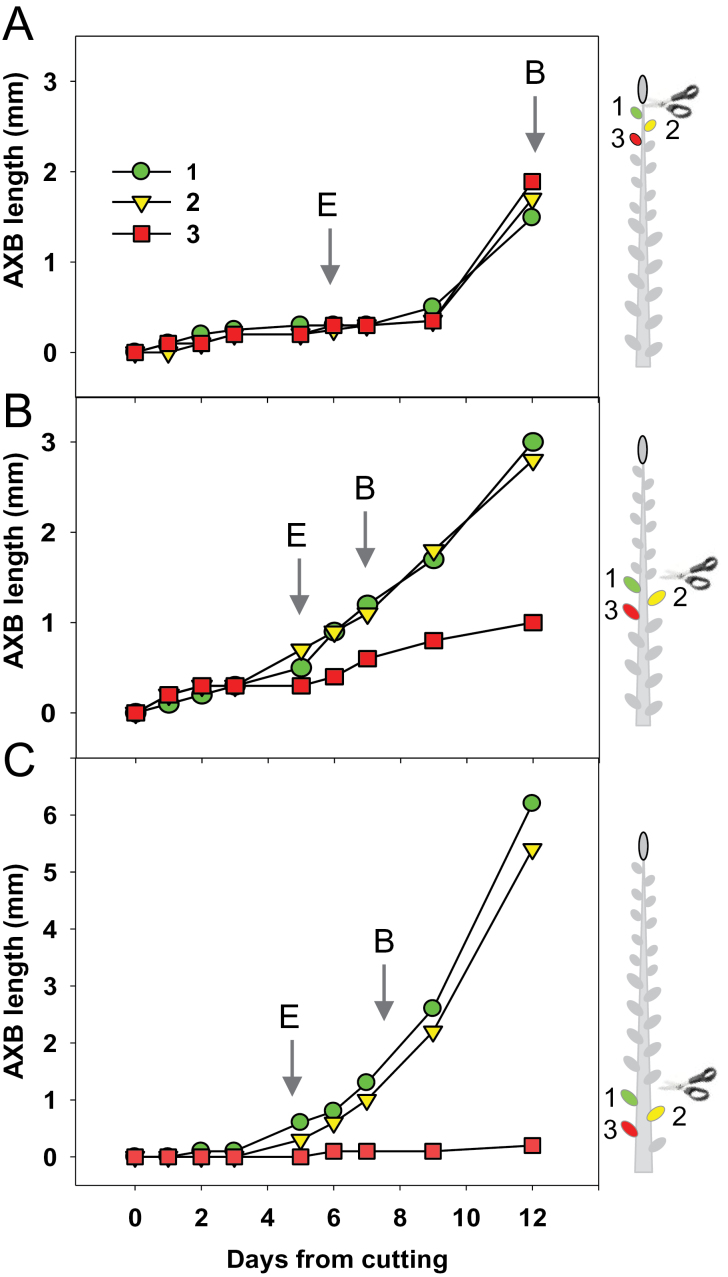
Decapitation-induced axillary bud (AXB) activation. Activation was assessed by measuring AXB elongation and burst. Stems were cut immediately under the apex (A), at the bud maturation point (BMP) (B), and at a lower position among mature AXBs (C) as illustrated in the schemes on the right (leaves are not depicted). The arrow marks the day when AXBs had significantly enlarged (E) (*n*=5; *P*<0.01; Student’s *t*-test), and the arrow ‘B’ when AXBs burst.

In the proleptic hybrid aspen clone T89, all branching from AXBs is absent during the first growing season, regardless of their maturation level, and even in the following season branching is restricted to a subset of AXBs. Nonetheless, first-season para-dormant AXBs can be activated by decapitation. To assess if AXB activation depends on the developmental status of the enclosed ES, plants were decapitated at various heights of the stem. This showed that, eventually, all AXBs are able to elongate and burst, but that the time required for activation is different (Fig. 4). The youngest AXBs needed an extended period of up to 2 weeks before bud burst was detectable (Fig. 4A), probably reflecting the time required to advance internal development. This suggests that a certain minimum stage of maturity (i.e. ES development) is required before bud elongation can take place. In contrast, AXBs at or below the BMP burst within a single week (Fig. 4B, C). Additional ageing of AXBs did not further advance the timing of bud burst, although older AXBs commonly produced faster growing branches (Fig. 4; Supplementary Fig. S1 at *JXB* online).

### AXBs above the BMP are responsive to short days

Photoperiodic signals that are generated in the leaves are involved in triggering developmental transitions at the apex. Growing apices are strong sinks in which cellular proliferation is fuelled by sugars and nitrogenous compounds imported via the phloem. Simultaneously, phloem sap delivers mobile signals that redirect development, among others the signal peptide FLOWERING LOCUS T (FT) and a host of small RNAs. The AXBs above the BMP are also sinks, because they are actively producing cells and tissues for the developing ES, albeit that cell stretching is absent. This raised the question of whether, and to what degree, AXBs are susceptible to phloem-delivered signals, and if this would influence their ability to establish dormancy under short days. To address this experimentally, plants were exposed to a dormancy-inducing short-day regime, and subsequently the burst capacity of AXBs was tested at various stages of development and ageing. In the long-day conditions used here, the apex harbours ~10 leaf primordia, each with an AXM. During exposure to a short photoperiod, they produced AXBs that occupied positions 1–10 (Supplementary Fig. S2 at *JXB* online). For bud burst testing, this cluster was divided into two groups containing AXB 1–5 and AXB 6–10 (Table 1). The ~10 visible AXBs between the apex and the BMP were subdivided into group 1 and 2. After a short photoperiod, the AXBs of these groups occupied positions 11–15 and 16–20, respectively. The 10 AXBs directly below the BMP represented a mature and an ageing group (group 3 and 4, respectively). After a short photoperiod, the AXBs of these groups occupied positions 21–25 and 26–30, respectively (Table 1; Supplementary Fig. S2 at *JXB* online). For each category, the dormancy status of AXBs was assessed in single-node cutting tests under growth-promoting conditions (Table 1; Rinne *et al.*, 2011). A pronounced negative correlation was consistently found between AXB maturity and the capacity to establish a dormant state (Table 1). The AXBs in the axils of the 10 youngest leaves (position 1–10) were dormant as they did not show any signs of burst, whereas the large majority of AXBs that in long days were clearly below the BMP (position 21–25) or in the ageing phase (position 26–30) did not establish dormancy. Above the BMP, most of the developing AXBs (position 11–15) established dormancy, but this capacity rapidly diminished during AXB completion toward the BMP (position 16–20). Together this showed that as a rule AXBs could establish dormancy during the phase of early development. Being a sink might thus be a major determinant of the capacity to establish dormancy.

**Table 1.** S*hort day (SD)-induced AXB dormancy* The upper AXBs (position 1–10) that developed under SDs from meristems in the axils of existing primordia all established dormancy. The older AXBs (position 21–30), which were at or below the bud maturation point (BMP) when SD started, had already completed their development, and the majority did not develop dormancy. AXBs in the middle positions (11–20) represents the AXBs above the BMP in long-day plants. The youngest of these AXBs, which were still developing when SDs started, mostly established dormancy (11–15), but this capacity diminished in AXBs closer to the BMP (16–20). The morphologically determined BMP of long-day plants, at which new primordia no longer emerge, was reached around AXB position 12 (*n*=10 plants). SD-induced dormancy was assessed by testing the bud burst capacity under growth-promoting conditions.

AXB position	Photoperiod during development	AXB burst %
1–5	SD	0
6–10	SD	0
11–15	LD/SD	38
16–20	LD/SD	90
21–25	LD	90
26–30	LD	100

Abbreviations: AXB, axillary bud; BMP, bud maturation point; ES, embryonic shoot; PATS, polar auxin transport stream; PD, plasmodesmata; SAM, shoot apical meristem; TB, terminal bud.

### Meristem-specific genes in AXB and TB development

The AXM, which produces an ES inside a developing AXB, is the equivalent of the SAM that produces an ES in the short-day-induced TB. The SAM and the AXM are actively engaged with primary morphogenesis so long as the ES is not complete. To map the expression of selected meristem identity genes during ES development, putative orthologues of the *Arabidopsis* genes *WUS*, *CLV1*, *CLV3*, and *KN1* were identified in the *P. trichocarpa* genome (Tuskan *et al.*, 2006), and their expression levels were analysed by qPCR.

**Fig. 5.**
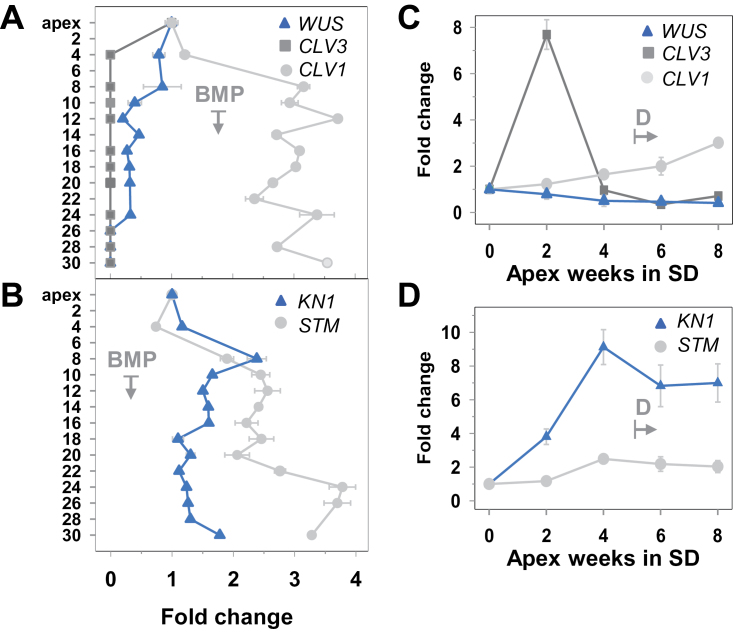
Expression analysis of genes involved in meristem organization and maintenance during axillary bud (AXB) and terminal bud (TB) development. Expression patterns (fold changes) of hybrid aspen (A and C) *WUSCHEL*-like (*WUS*), *CLAVATA 3*-like (*CLV3*), and *CLAVATA1*-like (*CLV1*), and (B and D) *KNOTTED1*-like (*KN1*) and *SHOOT MERISTEMLESS*-like (*STM*) genes. (A and B) Expression levels in the apex and AXBs in long days (C and D), and expression in the apex under short days (SD). T-shaped arrows mark AXBs below the bud maturation point (BMP) (A and B), and the time point of dormancy induction (D) in TBs (C and D). Values represent the means of six plants ±SE, analysed in two pooled samples.

In apices, the two identified *WUS*-like genes were expressed at a similar level. The gene with highest similarity to the *Arabidopsis WUS* gene (*WUSL1*; Supplementary Table S1 at *JXB* online) was selected for further analysis. *WUS* expression in AXBs was lower than in the apex, and was gradually down-regulated towards the BMP. In ageing AXBs (below node 24), transcript levels fell below the detection limit (Fig. 5A). In short-day-induced TBs *WUS* was moderately down-regulated (Fig. 5C), but less than in AXBs.

Blasting the *Arabidopsis CLV1* against the *P. trichocarpa* genome resulted in a large number of highly significant hits, but *CLV3* gave only three sequences with very low scores. A *CLV3*-like gene was selected on the basis of the presence of a C-terminus similar to the one in the *Arabidopsis CLV3*, and its presence in the shoot meristem library (http://popgenie.org/). The selected *CLV1* and *CLV3* genes were both expressed in the shoot apex of hybrid aspen. In developing AXBs, *CLV1* was up-regulated 3-fold, corresponding to the level found in short-day-induced TBs (Fig. 5A, C). In the apex, *CLV3* was expressed at a similar low level to *WUS*, whereas in all AXBs it was hardly expressed, or was below the detection limit (Fig. 5A). Under short days, *CLV3* expression was up-regulated in TBs after 2 weeks of short days, but down-regulated at week 4, and further until dormancy was established (Fig. 5C).

The expression levels of the two selected *KNOX* genes, with high similarity to *KN1* and *STM*, increased 2- and 3-fold during AXB development (Fig. 5B). While *STM* was further up-regulated during AXB ageing, *KN1* decreased in AXBs below the BMP to apex levels (Fig. 5B). During short-day-induced TB formation, both genes were up-regulated, particularly *KN1* (Fig. 5D). These patterns show that short-day-induced TBs and developing long-day AXBs share grossly similar gene expression trends, except for the transient expression peak in the putative *CLV3* gene during early TB development.

### Genes involved in SAM identity and branching

**Fig. 6.**
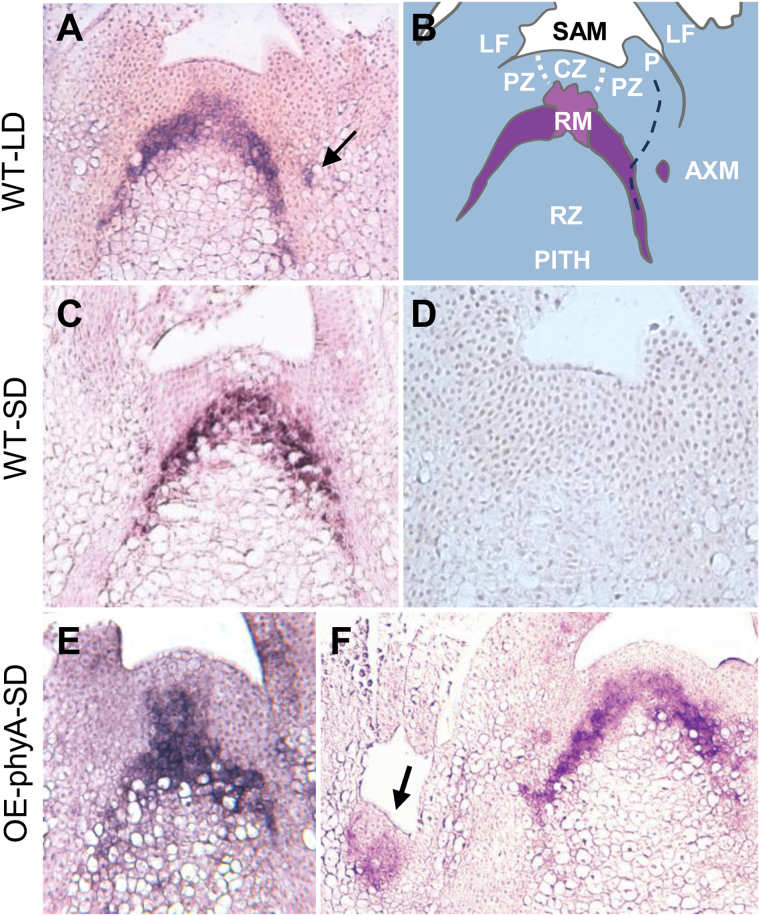
*In situ* hybridization of *CENTRORADIALIS-LIKE1 (CENL1*). (A–D) Expression patterns in the apex of wild-type (WT), and (E–F) transgenic hybrid aspen overexpressing oat phytochrome A (OE-phyA). (A) Long day (LD) apices show a bell-shaped expression domain, sheathing the uppermost part of the pith. Note *CENL1* expression in young axillary meristem (AXM, arrow). (B) Schematic depiction of the expression domain in (A). The upper part of the *CENL1* domain is the rib meristem (RM), immediately subjacent to the central zone (CZ). *CENL1* expression reaches to cell layer 7 or 6, which may be upper rib meristem or lower corpus. Leaf primordia (P), and the position of pro-vascular tissue (stippled). PZ, peripheral zone; LF, leaf; RZ, rib zone, where cell division and elongation occur simultaneously. (C) *CENL1* expression after 2 weeks of short days (SD). (D) Sense probe. (E) *CENL1* expression after 1 week of short days. The domain reaches 1–2 cell layers higher than in the WT. (F) Expression domain after 2 weeks in short days. Note expression in AXM (arrow).

In trees, *CENL1* is characteristically expressed in AXBs (Mohamed *et al.*, 2010), as well as in the apex and short-day-induced TBs prior to dormancy establishment (Ruonala *et al.*, 2008). As shown previously by qPCR, *CENL1* is expressed in an area that corresponds approximately to the rib meristem in the apex of hybrid aspen (Ruonala *et al.*, 2008). To establish this more precisely, the *CENL1* expression domain was mapped by *in situ* hybridization in long-day apices and developing short-day-induced TBs at the time point when its transcript levels are known to rise (Ruonala *et al.*, 2008). In the growing apex, *CENL1* was expressed specifically in the rib meristem and the early descendant cells that encircled the radially expanding pith. In sections this was visible as a bell-shaped domain (Fig. 6A, B). In the rib meristem, where radial expansion is still absent, *CENL1* was detected in a hat-shaped domain immediately subjacent to the SAM, and with the same width as the central zone of the meristem. *CENL1* was also detected very early in emerging AXMs (Fig. 6A, B). In short-day-induced TBs the hat-shaped domain faded, corresponding to the arrest of cell elongation in the rib meristem (Fig. 6C). That *CENL1* is involved in rib meristem activity is corroborated by observations on hybrid aspen lines that overexpress the oat *PHYA* gene. Whereas under long days the saplings are stunted, they accelerate internode elongation under short days, while the plastochron remains unchanged (Ruonala *et al.*, 2008). Here it is shown that this is accompanied by a pronounced hat-shaped *CENL1* expression domain (Fig. 6E). Although this was not visible in all sections (Fig. 6F), possibly relating to plastochron stage and rib meristem rhythmicity, it links enhanced rib meristem activity to expansion of the expression domain (Fig. 6E).

**Fig. 7.**
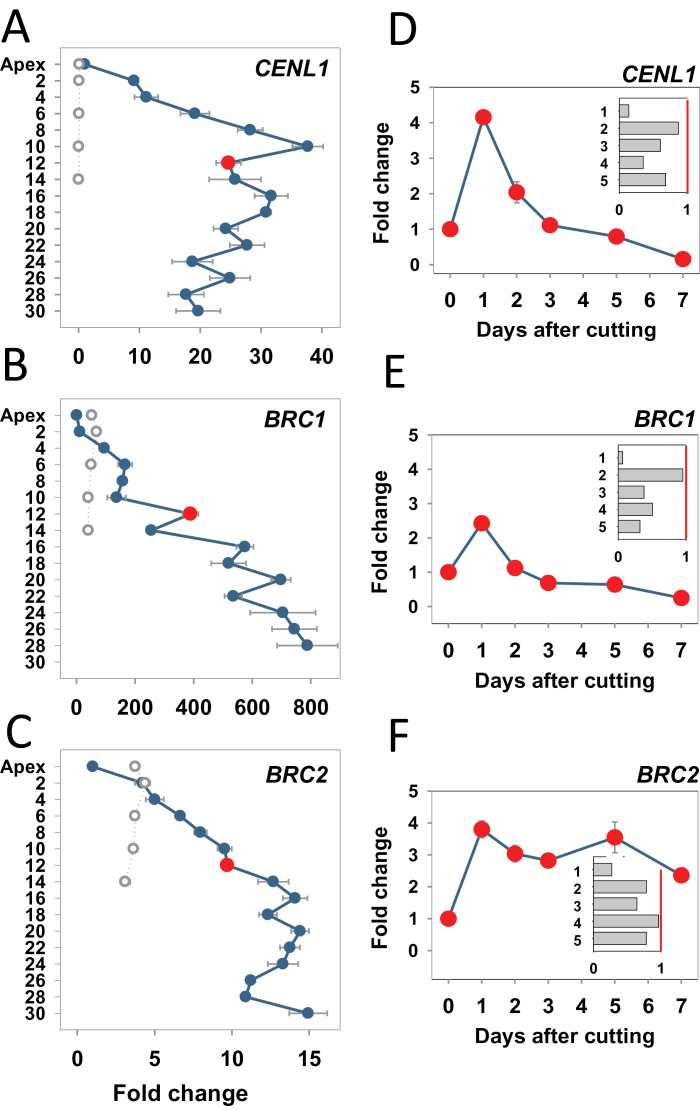
Expression analysis of genes involved in meristem identity and branching in plants grown under long and short photoperiods and after stem decapitation. (A and D) *CENTRORADIALIS-LIKE1* (*CENL1*); (B and E) *BRANCHED1-like* (*BRC1*); (C and F) *BRANCHED2-like* (*BRC2*). (A–C) Intact plants grown under long days (LD; blue dots and lines) and after 5 weeks under short days (SD; open circles, stippled lines). Long-day samples include the apex and axillary buds (AXBs) until node 30, and short-day samples include terminal buds and AXBs up to node 14. The red dot indicates the expression level (*x*-axis fold change) in AXB at node 12 of intact plants, the bud maturation point (BMP). (D–F) Stems were cut just above the BMP, and gene expression was followed for 7 d in AXB 12. Inserts indicate relative expression in five successive AXBs (position 1–5) proximal to the cut, 8 d after decapitation (*y*-axis fold change). Values in A–C are calculated relative to the apex (set at 1). Values in D–F are relative to AXB 12 of the intact plant (set at 1, red dot in A–C). Values in the insets of D–F are relative to each individual AXB position in the intact plant (set at 1, red line). Values represent the means of six plants ±SE, analysed in two pooled samples.

*CENL1* expression was also established by qPCR in apices and AXBs. In developing AXBs, transcript levels gradually rose to ~35-fold at the BMP, relative to the apex (Fig. 7A). Below the BMP, *CENL1* expression in AXBs was maintained at high levels. In strong contrast, in all AXBs that established dormancy, *CENL1* was completely down-regulated (Fig. 7A). Thus, maintenance of *CENL1* expression after bud completion characterizes para-dormancy and corresponds to a state that is poised for vegetative growth, whereas its complete down-regulation reflects dormancy establishment. Other phosphatidylethanolamine-binding protein family genes, *CENL2*, *FT1*, and *FT2* (Supplementary Table S1 at *JXB* online), were hardly expressed in buds (not shown). In contrast, the *P. trichocarpa* homologue of *BROTHER OF FT* (*BFT*) showed a gradual 8- to 10-fold up-regulation (Supplementary Fig. S3A at *JXB* online), a trend similar to that of *CENL1*. Expression levels of *BFT* in the dormant TB were elevated 4-fold, relative to the long-day apex. Under short days, AXBs increased *BFT* expression approximately to the same level as they would have done under long days, but at 5 weeks *BFT* expression was equalized between TBs and AXBs that established dormancy.

Two *TCP*-like genes, *BRC1* and *BRC2*, which are similar to the *Arabidopsis* branching-inhibiting genes, were identified in the *P*. *trichocarpa* genome (Supplementary Fig. S4 at *JXB* online). In long days, the expression levels of these genes increased considerably in developing AXBs, and even below the BMP in ageing AXBs (Fig. 7B, C). A 5-week short day exposure gradually induced significant increases in *BRC1* and *BRC2* levels also in TBs, ~50- and 4-fold, respectively. However, the values were lower than those of long-day AXBs at the BMP. Under short days, AXBs increased *BRC1* and *BRC2* levels to approximately the same level as they would have done under long days, but at 5 weeks the expression was equalized to the same level between TBs and AXBs.

### Hormone biosynthesis and signalling genes in TBs and AXBs

**Fig. 8.**
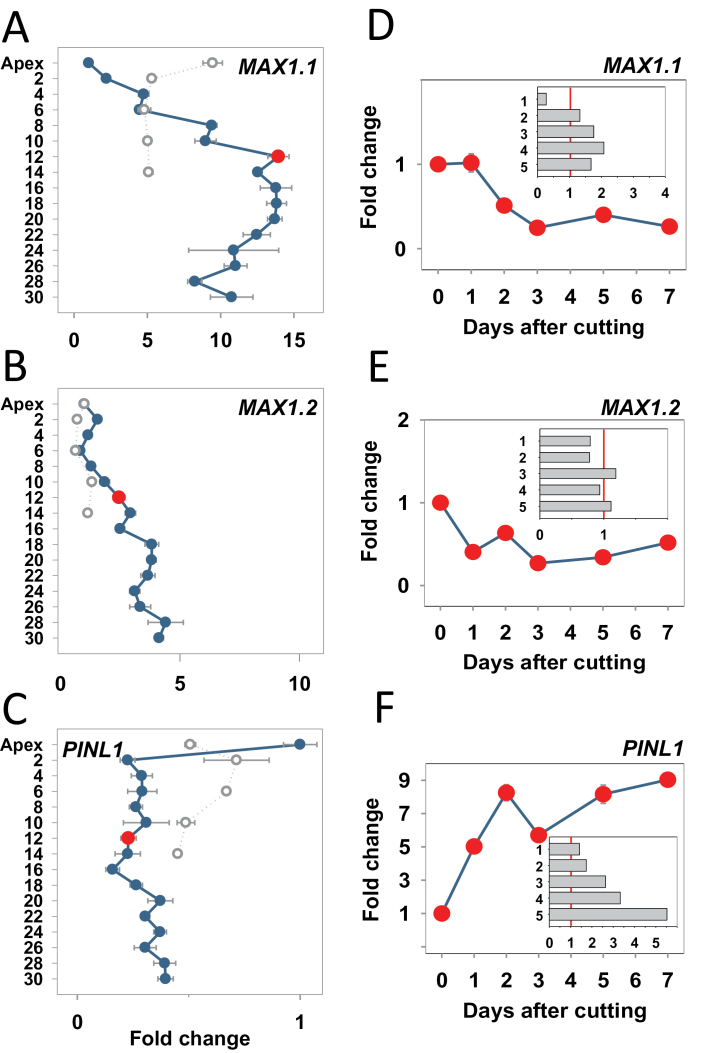
Expression analysis of genes of the strigolactone and auxin pathways in plants grown under long and short photoperiods and after stem decapitation. (A and D) *MORE AXILLARY BRANCHES1* (*MAX1.1*); (B and E) *MAX1.2*; (C and F) *PINFORMED1* (*PINL1*). (A–C) Intact plants grown under long days (LD; blue dots and lines) and for 5 weeks under short days (SD; open circles, stippled lines). Long-day samples include the apex and axillary buds (AXBs) until node 30 and short-day samples include the terminal bud and AXBs up to node 14. The red dot indicates the expression level (fold change on the *x*-axis) in AXB 12 of intact plants, the bud maturation point (BMP). (D–F) Stems were cut above the BMP and gene expression was followed for 7 d in AXB 12. Inserts indicate relative expression in five successive AXBs (1–5) proximal to the cut 8 d after decapitation (*y*-axis fold change). Values in A–C are calculated relative to the apex (set at 1). Values in D–F are relative to AXB 12 of intact plants (set at 1, red dot in A–C). Values in the insets of D–F are relative to each individual AXB position in the intact plants (set at 1, red line). Values represent the means of six plants ±SE, analysed in two pooled samples.

Hormones play a key role in AXB activation in herbaceous plants, but their role in perennial AXBs is less clear. The xylem-fed strigolactone analogue GR24 could inhibit the AXB burst, starting at a concentration of 0.5 μM (not shown), suggesting that endogenous strigolactone could be involved in inhibiting access of AXBs to the stem PATS. To assess the potential involvement of strigolactone and auxin in para-dormancy in hybrid aspen, the expression of a few *P. trichocarpa* genes, putatively belonging to these hormone pathways, were analysed. Two *Populus* genes were identified as homologues to the *Arabidopsis* putative strigolactone biosynthesis gene *MAX1* (Supplementary Fig. S5 at *JXB* online). These paralogues, named *MAX1.1* and *MAX1.2*, were both expressed in apices of hybrid aspen. In AXBs, the expression level of *MAX1.1* steadily increased towards the BMP, after which it remained relatively unchanged at a 10- to 12-fold level (Fig. 8A). *MAX1.2* expression was only slightly up-regulated when AXBs approached the BMP, but more strongly in the ageing AXBs below the BMP (Fig. 8B). They were also differentially expressed during dormancy. In dormant TBs, *MAX1.1* was strongly up-regulated, ~10-fold, approaching the expression levels of mature long-day AXBs (i.e. AXBs at the BMP and below) (Fig. 8A). The AXBs that matured under short days initially up-regulated *MAX1.1* to the long day level (not shown), but at 5 weeks in dormant AXBs, the level was lower than in TB (Fig. 8A). *MAX1.2*, in contrast, was not up-regulated in TBs. Although in AXBs developing under short days the *MAX1.2* expression was initially up-regulated (not shown), at dormancy both TBs and AXBs showed equal low expression levels (Fig. 8B).

To assess the possible involvement of auxin, two *Populus* genes with 73% and 65% identity to *Arabidopsis PIN1* auxin efflux carriers, named *PINL1* and *PINL2*, were selected. These genes, belonging to subclass-type 1 (Mravec *et al.*, 2009; Liu *et al.*, 2014), were both expressed in apices of hybrid aspen. Under long days, expression of *PINL1* and *PINL2* was about four times lower in developing para-dormant AXBs than in the growing apex (Fig. 8C; Supplementary Fig. S3B at *JXB* online). The low expression patterns of the *PINL1* and *PINL2* genes correspond to a lack of internode elongation in the ES, and may serve to inhibit branching. In ageing AXBs, below the BMP, their expression slightly increased. In short-day-exposed apices, expression of *PINL1* and *PINL2* was reduced within 2 weeks (not shown), whereas after 5 weeks they were further down-regulated in dormant TBs (Fig. 8C; Supplementary Fig. S3B at *JXB* online). In AXBs that developed under short days, the expression levels were up-regulated to a similar level to that in the TBs. The similarity in expression levels suggests that equalized expression levels may promote competition for access to the stem PATS, once dormancy is released and activation becomes possible.

### Decapitation changes gene expression in AXBs

As decapitation results in burst of AXBs and growth of the enclosed ES, it was investigated if this was preceded by changes in the expression of genes that are involved in branching, including *CENL1*, *MAX1.1*, *MAX1.2*, *BRC1*, *BRC2*, *PINL1*, and *PINL2*. To avoid measuring gene expression that is related to the developmental completion of the ES, stems were decapitated just below the BMP, at nodal position 12. Gene expression was first monitored over a 14 d period (not shown) and an 8 d period in five successive AXBs proximal to the cut (insets in Figs 7 and 8). This showed that only a few AXBs proximal to the cut substantially changed their gene expression pattern. This is congruent with the observation that, as a rule, decapitation only activated a few of the uppermost AXBs, giving rise to branches. Another experiment, with daily analyses of the proximal AXB only, showed that significant changes in gene expression took place already within the first day (e.g. Figs 7D–F, 8D–F). Enlargement of these already mature AXBs, a pre-stage of bud burst, became visible only later (Fig. 4).

*CENL1* expression, which in AXBs of intact plants increased toward the BMP, was up-regulated 4-fold 1 d after decapitation (Fig. 7D), but subsequently down-regulated to the level of growing apices (Fig. 7D, inset). This boost of *CENL1* expression prior to any visible enlargement of the AXBs may serve to activate the rib meristem. The subsequent lower *CENL1* expression levels, typical of the growing apex, may indicate that the former AXM had assumed the role of the SAM for the side shoot. The *BFT* gene, in contrast, was up-regulated during AXB development, maintained during para-dormancy, and down-regulated after decapitation in the proximal AXB (Supplementary Fig. S3B at JXB online). In four AXBs under the proximal AXB, expression levels of *BFT* were up-regulated, possibly reflecting the inhibition of multiple branch outgrowth.

*BRC1* and *BRC2* expression, which in AXBs of intact plants was up-regulated toward the BMP (Fig. 7B, C), was transiently further up-regulated after decapitation (Fig. 7E, F). *BRC1* was down-regulated 2 d after decapitation, before activation was detectable. In contrast, *BRC2* expression remained elevated longer until AXBs were visibly elongating at day 8 (Fig. 7F, inset), suggesting that it does not function early in the branching process.

Expression of *MAX1*-like genes was under complex regulation in AXBs after decapitation. Expression of *MAX1.1*, which increased during AXB development and was maintained at maturity (Fig. 8A), decreased significantly in the proximal AXB 1 d after decapitation, and before AXB elongation was detectable (Fig. 8D). While the low expression level was maintained after 8 d, the AXBs at a lower position had slightly increased expression levels (Fig. 8D, insert), possibly reflecting the inhibition of multiple branch outgrowth, as in the case of *BFT* (Supplementary Fig. S3 at *JXB* online). Decapitation resulted in a rapid reduction of *MAX1.2* expression in the AXB closest to the cut, while the two uppermost buds maintained slightly reduced levels even at 8 d post-decapitation (Fig. 8E, inset).

Although in para-dormant AXBs of intact plants *PINL1* and *PINL2* were little expressed (Fig. 8C), they were significantly up-regulated 1 d after decapitation in the AXBs proximal to the cut, and further during subsequent days (Fig. 8F; Supplementary Fig. S3B at *JXB* online), reflecting the branching process. In independent sets of experiments expression levels in the upper AXB returned to moderate values 8 d post-decapitation (Fig. 8F, inset; Supplementary Fig. S3, inset at *JXB* online).

## Discussion

**Fig. 9.**
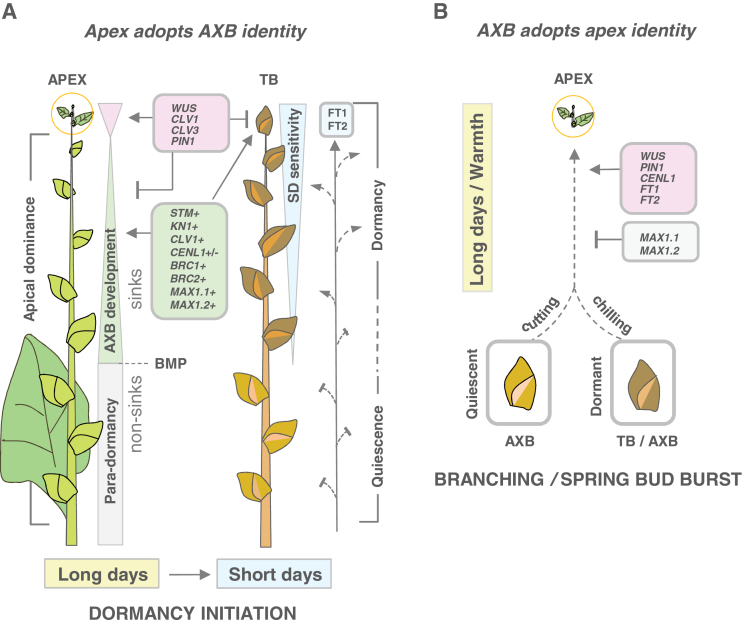
Conceptual model of the interactive environmental and molecular regulation of identity swapping of apices and axillary meristem in hybrid aspen. (A) In long days (LD), the shoot apical meristem (SAM), seated at the apex (encircled), produces internodes, leaves, and para-dormant AXBs. Under short days (SD), the SAM adopts axillary bud (AXB) identity and produces a terminal bud (TB). SAM-specific gene expression is down-regulated in developing AXBs as well as in developing TBs (pink triangle and pink gene box). During development, AXBs and TBs produce an embryonic shoot, while up-regulating bud-specific genes (green triangle and green gene box). Development is complete at the bud maturation point (BMP; stippled line), after which gene expression remains relatively stable (grey square). Key genes for SAM function and AXB development are mutually exclusive (opposing triangle summits; pink and green gene boxes; + indicates up-regulation, +/– up-/down-regulation). AXBs keep the same basic programme in both photoperiods, but after 5 weeks of SDs in both AXBs and TBs *CENL1* is completely down-regulated during dormancy establishment. AXBs gradually lose their responsiveness to SDs (inverted blue triangle) towards the BMP, where sink activity and accessibility to phloem-delivered photoperiodic signals such as FT1 and FT2 have ceased (blue peptide box, arrows). These AXBs overwinter in a suppressed, quiescent state. AXB number is arbitrary. (B) During bud burst and branching, AXBs adopt apex identity. This requires release from dormancy by chilling and LDs/warmth, or removal of para-dormancy by cutting. Genes that promote development of second-generation apices (pink gene box) are shared with the apex, and genes that oppose this (grey gene box, T-shape) are shared with AXBs.

The genetic and physiological parameters that govern plant architecture are conserved among angiosperms (Wang and Li, 2006). Nevertheless, growth habits are so variable that one might expect that branching mechanisms have diversified, reflecting variable implementation of conserved branching processes. For example, the rosette growth habit of *Arabidopsis* is very different from the caulescent growth form of pea. On another level, woody perennials are vastly different from both annuals, and unique regulatory principles must operate to account for the presence of juvenile and adult stages (Brunner *et al.*, 2014) as well as a seasonal dormancy cycle (van der Schoot and Rinne, 2011). To explore this unknown territory, AXB and TB development was mapped in hybrid aspen, *Populus* orthologues of relevant *Arabidopsis* genes were identified, and their expression patterns were analysed in AXBs during their development and during decapitation-induced activation and outgrowth. For the sake of experimental simplicity, the investigations were carried out with first-year saplings that were grown under controlled conditions. Moreover, because in first-year saplings the AXBs are repressed by apical dominance (Cline *et al*., 1997), a comparison with branching in annuals is a realistic possibility. Nonetheless, distinct differences were found related to AXM initiation, and AXB development, structure, and composition. The findings are summarized in a model (Fig. 9).

### AXBs and TBs of hybrid aspen are perennating structures

In hybrid aspen, AXMs arise in the axils of all emerging leaves, and immediately start forming bud scales and an ES (Figs 1, 2). As a result, a range of different developmental stages is present along the elongating parent stem (Fig. 9). Because as a rule AXBs remain inhibited, changes in gene expression reflect exclusively AXB development, and not the sudden activation of AXBs. Although in the rosette plant *Arabidopsis* the initiation of AXMs starts in the axils of lower rosette leaves (Grbic and Bleecker, 2000; Long and Barton, 2000; Greb *et al.*, 2003), the developmental gradient of AXBs has a similar orientation. In both cases, the more developed AXBs are the lowest on the stem. Nonetheless, the AXBs in hybrid aspen are very different from those in the annual *Arabidopsis*. In *Arabidopsis*, the term ‘AXB’ denotes an AXM that has produced a couple of small leaves but no scales, possibly resembling the incomplete AXBs that give rise to the sylleptic branches of woody perennials (Wu and Hinckley, 2001). The perennating AXBs of the proleptic hybrid aspen clone T89 are sturdy structures with hardening scales that form irrespective of photoperiod to serve as protective devices for the ES in winter (Figs 1, 2, 9) (van der Schoot *et al.*, 2014).

Unique to woody perennials is that the SAM transforms under short days into a perennating structure that is similar to an AXB (Figs 1, 2, 9). Interestingly, the SAM adopts the developmental programme of its daughter meristems, not only structurally, but also in terms of how they regulate *BRC1*, *BRC2*, and *MAX1.1* (Figs 7, 8). Although scales of TBs and AXBs are formed differently, corresponding to their different origin (Figs 2, 3), they are identical in function. In both cases, bud formation requires switches in the identity of meristem primordia: in TBs from leaf-to-scale-to-embryonic leaf identity, and in AXBs from scale-to-embryonic leaf identity. These tightly regulated processes are crucial underpinnings of the perennial lifestyle.

### Redefining para-dormancy in hybrid aspen

In the dormancy literature, absence of branching is often referred to as para-dormancy, to distinguish it from dormancy. More specifically, it may be defined as the suspension of axial development and growth imposed by other plant parts (reviewed in Atwell *et al.*, 1999; van der Schoot and Rinne, 2011). The term correlative inhibition may refer to the same phenomenon, denoting suppression of AXB outgrowth, usually by an actively proliferating apex. The literature is ambivalent about their precise meaning, and both terms may refer to absence of branch formation, or to AXBs that either grow slowly or not at all. The present data suggest that for hybrid aspen the term para-dormancy can be defined in a specific way. Although none of the AXBs bursts, only those below the BMP suspend axial growth and development. Up to that point, AXBs show considerable developmental activity, even if the shoot is compressed and shielded from vision. They produce a tightly packed rosette of ~10 embryonic leaves (Figs 1, 2, 9) in a period as short as 4 weeks. In the same period, the SAM had produced ~10 younger phytomers. The developmental activity of the SAM/apex and the AXM/AXB thus appears quite comparable. In conclusion, the ES (Romberger, 1963; van der Schoot *et al.*, 2014), or ‘pre-formed shoot’ (Brunner *et al.*, 2014), is a genuine and complete shoot, albeit with unextended internodes and unexpanded leaves. It is in some ways reminiscent of a rosette growth form (Romberger, 1963). While elongation of this ES is prevented by apical dominance, its development continues up to the BMP. Shoot elongation at the apex is similarly prevented under short days. Thus, there appears to be a direct parallel between AXB and TB formation. In TBs, ES completion is followed by dormancy establishment, whereas in AXBs it is followed by para-dormancy under long days and, when completed under short days, by dormancy (Fig. 9).

Developing AXBs (above the BMP) and para-dormant AXBs (at and below the BMP) were also functionally distinct. They differed in the ease with which they produced a branch upon decapitation (Fig. 3), and in their capacity to establish dormancy (Table 1). The AXBs above the BMP, emerging TBs, and apices, are all supplied by the phloem. They import not only sugars and nitrogenous compounds, but also photoperiodic signals (Fig. 9; Sachs, 1991). To some degree this is even true in *Arabidopsis*, where FT imported in AXBs is neutralized by BRC1 (Niwa *et al.*, 2013). The strongly diminished capacity to establish dormancy in AXBs around and below the BMP (Table 1) might therefore reflect cessation of sink activity. In brief, AXBs above the BMP resemble developing TBs more than the para-dormant AXBs below the BMP.

### CENL1 *is a rib meristem-identity gene*

In hybrid aspen, *CENL1* is expressed in growing apices (Ruonala *et al.*, 2008) as well as in AXBs (Fig. 7A). Expression in AXBs was reported previously for species as distinct as tomato and *Populus* (Pnueli *et al.*, 1998; Mohamed *et al.*, 2010). The current data show that *CENL1* is considerably up-regulated during AXB development, and that the highest expression levels are reached in para-dormant AXBs at and below the BMP (Fig. 7A). Up-regulation characterizes bud formation as such, as *CENL1* is also transiently up-regulated in short-day-induced TBs, prior to dormancy establishment (Ruonala *et al.*, 2008). The *in situ* hybridizations show that the *CENL1* expression domain is located at the rib meristem (Fig. 6), confirming earlier qPCR-based estimates for apices (Ruonala *et al.*, 2008).

That *CENL1* expression is required for rib meristem activity is concluded from the following facts. Hybrid aspen saplings that overexpress the oat *PHYA* gene, which reduces stem elongation (Jordan *et al.*, 1995), are stunted. However, under short days these transgenic plants up-regulate *CENL1* and increase elongation (Ruonala *et al.*, 2008), while concomitantly expanding the *CENL1* expression domain at the rib meristem (Fig. 6E). It is proposed, therefore, that in hybrid aspen *CENL1* functions as a rib meristem-identity gene. The rib meristem may not only support stem elongation by supplying cells (Rinne *et al.*, 2005), but it may also function as a putative signal relay station between the stem and SAM (Ruonala *et al.*, 2008; Paul *et al.*, 2014). During AXB and TB formation, cell divisions in the rib meristem continue, but cell stretching is virtually absent, enforcing the dwarfed stature of the ES. This is accompanied by a steady increase in the level of *CENL1* expression (Fig. 7A), suggesting that up-regulation is needed to support the completion of the ES before the inhibiting forces that promote para-dormancy take the upper hand.

### *Does* CENL1 *keep AXBs poised for vegetative growth?*

In AXBs below the BMP, the expression levels of *CENL1* are kept high (Fig. 7A), possibly keeping these para-dormant AXBs poised for growth. This conjecture is supported by correlative evidence. First, preparedness for growth is lost when AXBs and TBs down-regulate *CENL1* during dormancy establishment. Secondly, decapitation triggers AXB activation and branching in *CENL1*-expressing cells, but not in dormant AXB/TBs that have shut off *CENL1*. Thirdly, during the first 2–3 weeks of short-day-induced TB formation, when *CENL1* expression is up-regulated, reversal to normal growth is possible, but not at later stages, when the gene is switched off (Ruonala *et al.*, 2008). Lastly, *CENL1* expression is boosted immediately after decapitation, to return subsequently to levels representative of the growing apex (Fig. 7A). The proposed *CENL1* function is congruent with the functions of its orthologue *TFL1* in *Arabidopsis*. *TFL1* overexpressors have highly branched inflorescences and delayed flowering (Ratcliffe *et al.*, 1998). In contrast, *tfl* mutants are deficient in secondary inflorescences and accelerate flowering (Bradley *et al.*, 1997).

Unlike the sylleptic branching style, which is affected by growth vigour and environmental conditions (Ceulemans *et al.*, 1990; Wu and Settler, 1998), prolepsis is a stable branching style. Why then would a proleptic genotype, in which branching is delayed to the next growing season, keep AXBs poised for growth? It seems likely that the readiness of AXBs to grow out may be a strategy to deal with incidental damage to the apex, or adverse weather conditions during the growing season. Because in sylleptic genotypes branching from current-year AXBs can proceed without decapitation, its regulation might involve partly different mechanisms, although this remains to be investigated. Briefly, the up-regulation of *CENL1* at the rib meristem during ES formation supports the hypothesis that it is required to sustain cell divisions, and counteract increasingly strong inhibitory forces to maintain the preparedness of AXBs for growth.

### Are *BRC1* and *BRC2* counteracting *CENL1*?

Factors that could potentially counterbalance *CENL1* include *BRC* genes, which repress branching in *Arabidopsis* (Aguilar-Martínez *et al.*, 2007; Finlayson, 2007; Niwa *et al.*, 2013). This is supported by the fact that the *Populus* orthologues *BRC1* and *BRC2* were up-regulated during AXB formation and short day induction of TBs (Fig. 7B, C). Below the BMP, the expression of *BRC1*, possibly the main inhibitor of branching, continues to increase (Fig. 7B, C), whereas *CENL1* expression levels off (Fig. 7A). This could suppress further cell division activity at the rib meristem and halt ES development. Other inhibitory factors positively correlate with AXB inhibition, including hormones such as ABA, ethylene (Ruttink *et al.*, 2007), and strigolactone (Booker *et al.*, 2005; Brewer *et al*., 2009, 2013). Indeed, the data support that *MAX1.1*-mediated strigolactone production might counterbalance *CENL1* during AXB formation and para-dormancy (Figs 7A, 8A).

An additional role for *BRC1* and *BRC2* could parallel a function of *CENL1*, which is to safeguard the vegetative status of the AXBs. TFL1 can interact with FD in nuclei (Hanano and Goto, 2011) and compete with FT for binding to an FD–14-3-3 receptor complex (Conti and Bradley, 2007; Jaeger *et al.*, 2013). Up-regulation of the *TFL1* orthologue *CENL1* in para-dormant AXBs of hybrid aspen might thus help to diminish the change of floral induction. *BRC1* and *BRC2* might have a similar role in suppressing flowering in AXBs. In *Arabidopsis*, BRC1 selectively binds and neutralizes FT that is imported into the AXB, thereby suppressing floral transition (Niwa *et al.*, 2013). Although in adult *Populus* trees *FT1* induces flowering in a subset of AXBs (Böhlenius *et al.*, 2006; Hsu *et al.*, 2011; Brunner *et al.*, 2014), saplings of hybrid aspen do not flower despite the fact that chilling-induced release from dormancy hyper-induces *FT1* (Rinne *et al.*, 2011; Paul *et al.*, 2014). Although it is not known if chilling up-regulates *BRC1* and *BRC2* in hybrid aspen, the genes are up-regulated in the apex and AXBs during dormancy establishment (Fig. 7B, C). In spring, the TB and most AXBs burst, suggesting that equalization of *BRC1* and *BRC2* expression may safeguard indeterminacy and competitiveness in the race to become a leading shoot.

### *AXB behaviour and* PIN1 *and* MAX1 *genes*

Auxin has an important role in the initiation of leaf primordia at the SAM (Reinhardt *et al.*, 2003), and it is likely also to be involved in ES formation in AXBs and TBs. However, its polar transport toward the stem PATS might be prevented as *PIN1* levels are low in both AXBs and TBs, and branching and apical expansion are inhibited. When released from apical dominance by decapitation, the para-dormant AXBs just above the BMP increased the expression of *PINL1* and *PINL2* (Fig. 8C; Supplementary Fig. S3B at *JXB* online), the *Populus* orthologues of class I *PIN1* in *Arabidopsis* (Liu *et al.*, 2014). For example, *PINL1* expression increased to 9-fold, temporarily exceeding apex levels by >2-fold (Fig. 8C). Such an increase is congruent with data of Liu *et al.* (2014) showing that *PINL1* and *PINL2* (referred to as *PIN1a* and *PIN1c*) were up-regulated >5-fold during shoot regeneration in *Populus*. The low expression levels of *PINL1* and *PINL2* in almost all AXBs reflects apical dominance, as decapitation resulted in up-regulation in the AXBs proximal to the cut (Fig 8C; Supplementary Fig. S3B). Interestingly, short-day exposure enhanced *PINL1* and *PINL2* expression in AXBs, while somewhat lowering it in TBs (Fig 8C; Supplementary Fig. S3B). This equalization makes sense in a strategy in which all buds, once released from dormancy, have similar changes to initiate growth and access the stem supply routes.

As the genes for strigolactone biosynthesis are conserved across many species, including willow and *Populus* (Challis *et al.*, 2013; Ward *et al.*, 2013; Czarnecki *et al.*, 2014), their function might be widely shared. The strong up-regulation of *MAX1.1* during AXB development in hybrid aspen (Fig. 8A) may therefore be an important factor in inhibition of branching at current-year AXBs. That *MAX1.2* expression reached the highest levels in AXBs below the BMP may help to counteract the greater likelihood of burst in older AXBs where the ES is completed (Fig. 8B). Application of strigolactone causes rapid depletion of PIN1 proteins from the plasma membrane in *Arabidopsis* and pea, resulting in a loss of PATS (Balla *et al.*, 2011; Shinohara *et al.*, 2013; Waldie *et al.*, 2014). Given the conserved nature of *PIN1* and *MAX1* genes, and their role in annuals, the combination of low *PINL1*/*PINL2* expression (Fig. 8C; Supplementary Fig. S3B at *JXB* online) and elevated *MAX1.1/MAX1.2* expression (Fig. 8A, B) is likely to contribute to the inhibition of branching in hybrid aspen.

The change in expression of *MAX1.1* and *MAX1.2* in apex/TB and AXBs, during the switch from long days to short days, was opposite compared with that of the *PIN1* genes, but their expression was similarly equalized. This provides further support for the hypothesis that equalized gene expression facilitates the synchronized bud burst in spring, prior to the establishment of apical dominance by a leader shoot. This strategy could serve to spread risk among different shoots, allowing initial competition between the apex and apical branches.

In hybrid aspen and other woody perennials, MAX1-regulated strigolactone production might have an unexpected additional role, absent in herbaceous species. *MAX1.1*, but not *MAX1.2*, was highly expressed during short-day-induced dormancy in TBs as well as the uppermost AXBs (Fig. 8A, B). Since strigolactone typically controls AXB behaviour, the surprising finding that short days induced expression of the *MAX1.1* gene in the TB supports the hypothesis that under short days the apex adopts an AXB-like identity. Notably, both *MAX* genes were expressed by AXBs, implying that they produce strigolactone themselves, instead of relying on import (Ruyter-Spira *et al.*, 2013; Cavar *et al.*, 2014). Possibly, the production of strigolactone by AXBs is an adaptation enforced by a continuously expanding shoot system.

## Conclusions

Structural analyses and gene expression data suggest a conceptual model in which TBs and AXBs, despite being formed under different conditions in subsequent seasons, share overall development, structure, activation, and marker gene expression (Fig. 9). Under short days, the apex adopts the developmental trajectory of AXBs. TBs and developing AXBs are photoperiod-responsive sink organs, which establish dormancy after having completed their ES under short days (Fig. 9A). Under long days, branching is prevented by apical dominance, and the AXBs enter para-dormancy once they are complete and cease to be sinks. Quiescent AXBs and the dormant TB/AXBs that are activated by decapitation and chilling, respectively, adopt the developmental programme of the apex (Fig. 9B). *CENL1* is a rib meristem-identity gene, whose activity is required for growth at the apex, developing buds, and branching. High *CENL1* expression levels keep AXBs poised for growth, but are counterbalanced by *BRC1* and *BRC2*. Branching is further impeded by local strigolactone biosynthesis and repressed access to the stem PATS, as suggested by the contrasting and decapitation-reversible expression patterns of the studied *MAX1* and *PIN1*-like genes.

## Supplementary data

Supplementary data are available at *JXB* online.

Figure S1. Decapitation effects on AXB branching.

Figure S2. *A priori* defined categories of AXM/AXB.

Figure S3. *BFT* and *PINL2* expression.

Figure S4. Phylogenetic analysis of TB/BRC-like proteins.

Figure S5. Phylogenetic analysis of MAX1-like proteins.

Table S1. *P. trichocarpa* genes, identifiers, and primer pairs.

Supplementary Data
