# Supplementary material for: Long and short photoperiod buds in hybrid aspen share structural development and expression patterns of marker genes
Source: J Exp Bot. 2015 Aug 5;66(21):6745–60. doi: 10.1093/jxb/erv380 (PMC4623686; doi:10.1093/jxb/erv380)
Supplement: Supplementary Data [file supp_66_21_6745__index.html]

Long and short photoperiod buds in hybrid aspen share structural development and expression patterns of marker genes — Long and short photoperiod buds in hybrid aspen share structural development and expression patterns of marker genes — Supplementary Data 

# Long and short photoperiod buds in hybrid aspen share structural development and expression patterns of marker genes

## Supplementary Data

Data files

- Supplementary Data - Supplementary Data
- Supplementary Data - Supplementary Data
- Supplementary Data - Supplementary Data
- Supplementary Data - Supplementary Data
- Supplementary Data - Supplementary Data
- Supplementary Data - Supplementary Data
