# Supplementary material for: Long and short photoperiod buds in hybrid aspen share structural development and expression patterns of marker genes
Source: J Exp Bot. 2015 Aug 5;66(21):6745–60. doi: 10.1093/jxb/erv380 (PMC4623686; doi:10.1093/jxb/erv380)
Supplement: Supplementary Data [file supp_erv380_Supplementary_Fig._S1._legend.pptx]

## Slide 1
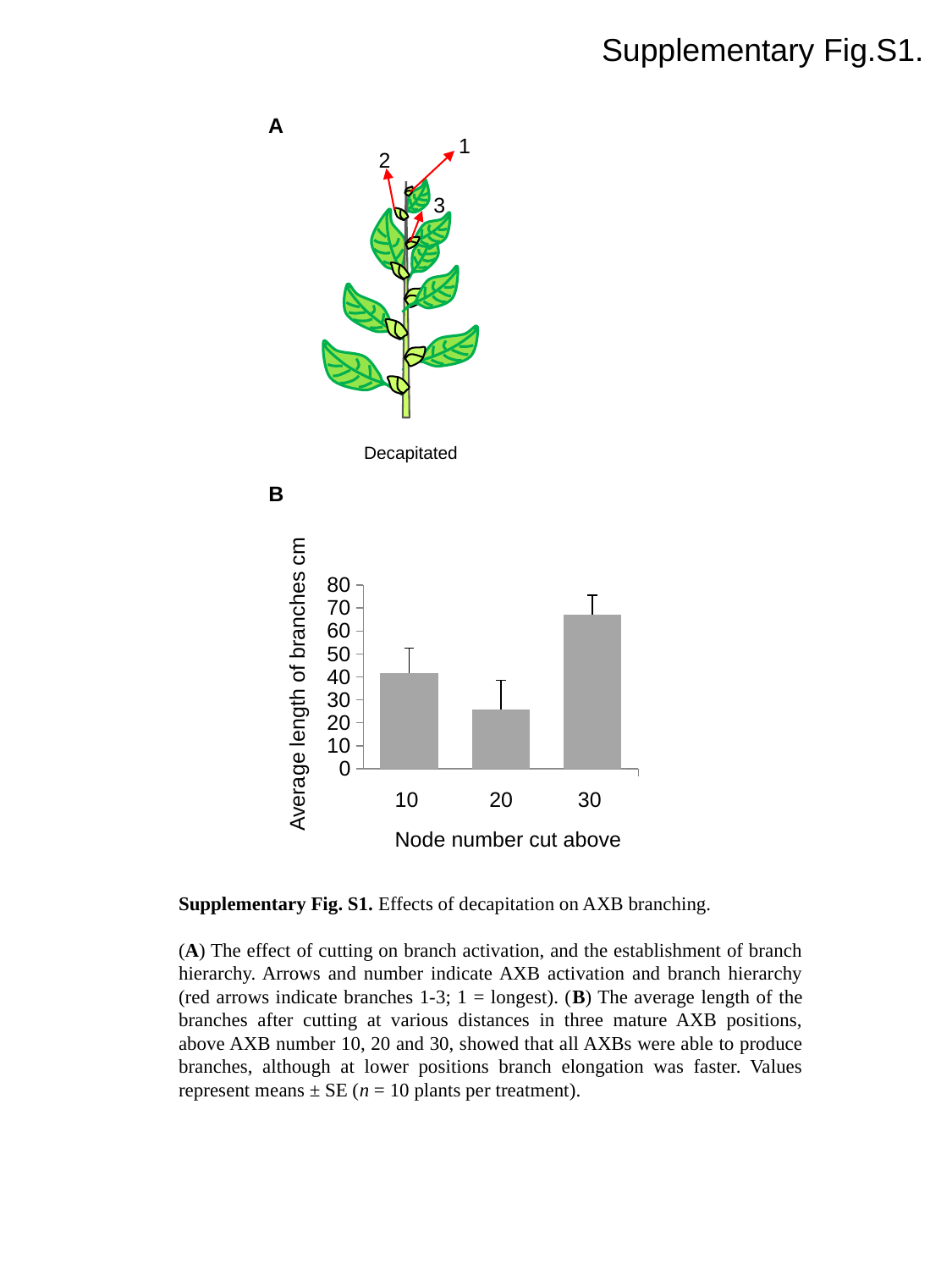

Supplementary Fig.S1.
A
1
2
3
 Decapitated
B
### Chart
| Category | |
|---|---|
| cut at AXB 10 | 41.82916666666659 |Average length of branches cm
 10 20 30
Node number cut above
Supplementary Fig. S1. Effects of decapitation on AXB branching.
(A) The effect of cutting on branch activation, and the establishment of branch hierarchy. Arrows and number indicate AXB activation and branch hierarchy (red arrows indicate branches 1-3; 1 = longest). (B) The average length of the branches after cutting at various distances in three mature AXB positions, above AXB number 10, 20 and 30, showed that all AXBs were able to produce branches, although at lower positions branch elongation was faster. Values represent means ± SE (n = 10 plants per treatment).
