# Supplementary material for: Long and short photoperiod buds in hybrid aspen share structural development and expression patterns of marker genes
Source: J Exp Bot. 2015 Aug 5;66(21):6745–60. doi: 10.1093/jxb/erv380 (PMC4623686; doi:10.1093/jxb/erv380)
Supplement: Supplementary Data [file supp_erv380_Supplementary_Fig._S3._legend.pptx]

## Slide 1
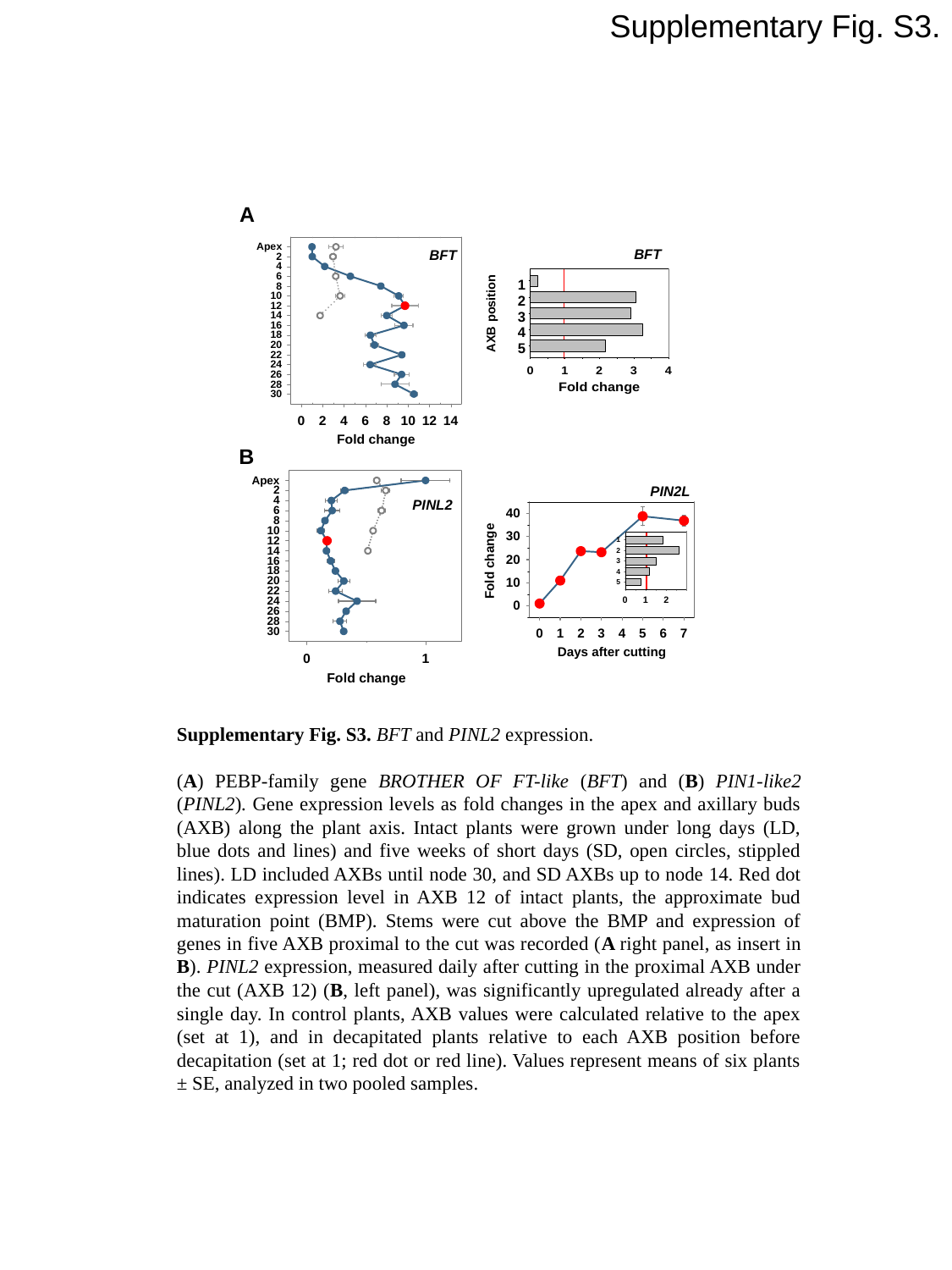

Supplementary Fig. S3.
A
BFT
1
2
3
4
5
AXB position
BFT
B
 PIN2L
1
2
3
4
5
 PINL2
Supplementary Fig. S3. BFT and PINL2 expression.
(A) PEBP-family gene BROTHER OF FT-like (BFT) and (B) PIN1-like2 (PINL2). Gene expression levels as fold changes in the apex and axillary buds (AXB) along the plant axis. Intact plants were grown under long days (LD, blue dots and lines) and five weeks of short days (SD, open circles, stippled lines). LD included AXBs until node 30, and SD AXBs up to node 14. Red dot indicates expression level in AXB 12 of intact plants, the approximate bud maturation point (BMP). Stems were cut above the BMP and expression of genes in five AXB proximal to the cut was recorded (A right panel, as insert in B). PINL2 expression, measured daily after cutting in the proximal AXB under the cut (AXB 12) (B, left panel), was significantly upregulated already after a single day. In control plants, AXB values were calculated relative to the apex (set at 1), and in decapitated plants relative to each AXB position before decapitation (set at 1; red dot or red line). Values represent means of six plants ± SE, analyzed in two pooled samples.
