# Supplementary material for: Long and short photoperiod buds in hybrid aspen share structural development and expression patterns of marker genes
Source: J Exp Bot. 2015 Aug 5;66(21):6745–60. doi: 10.1093/jxb/erv380 (PMC4623686; doi:10.1093/jxb/erv380)
Supplement: Supplementary Data [file supp_erv380_Supplementary_Fig._S2._legend.pptx]

## Slide 1
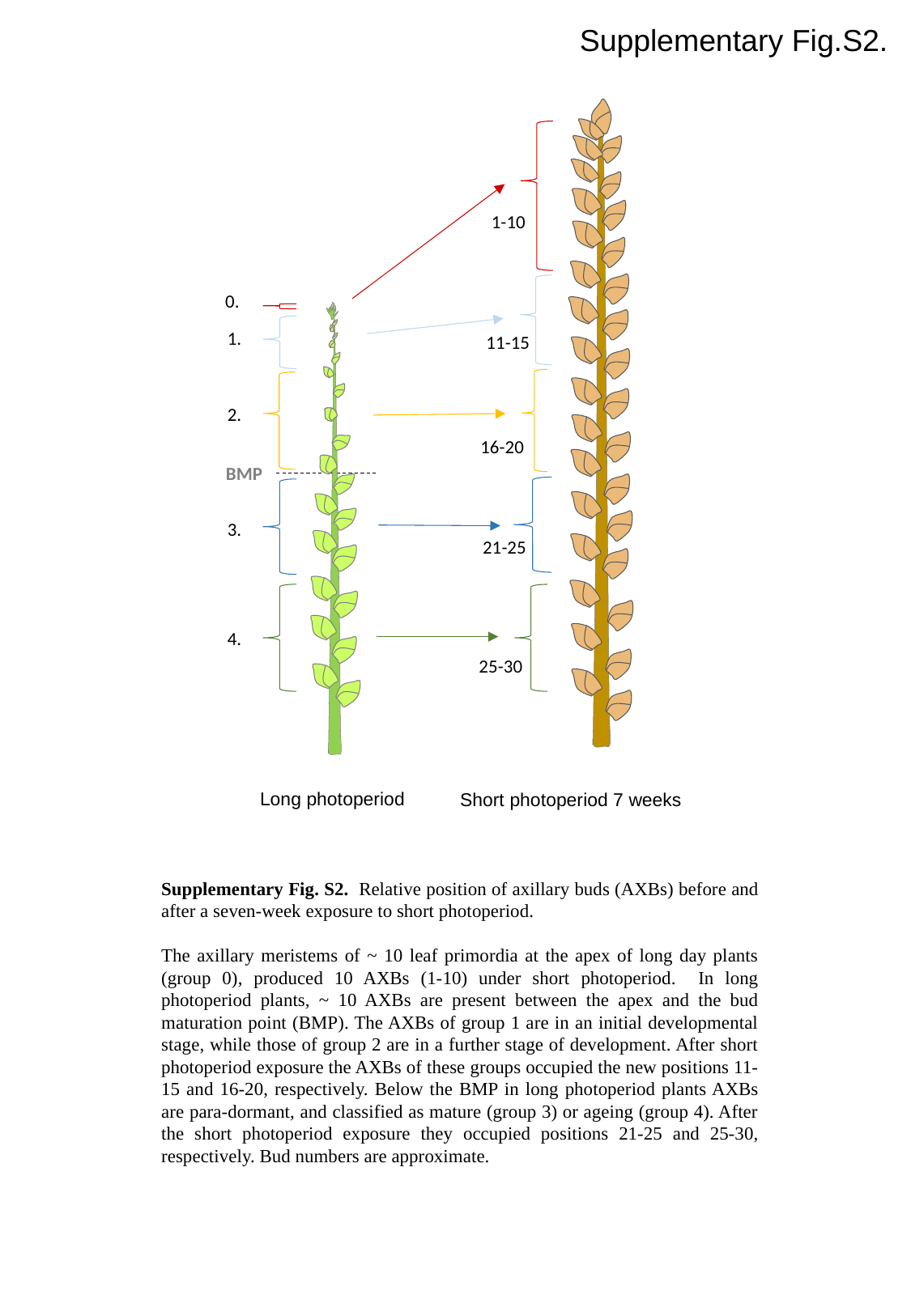

Supplementary Fig.S2.
1-10
0.
1.
11-15
2.
16-20
BMP
3.
21-25
4.
25-30
Long photoperiod
Short photoperiod 7 weeks
Supplementary Fig. S2. Relative position of axillary buds (AXBs) before and after a seven-week exposure to short photoperiod.
The axillary meristems of ~ 10 leaf primordia at the apex of long day plants (group 0), produced 10 AXBs (1-10) under short photoperiod. In long photoperiod plants, ~ 10 AXBs are present between the apex and the bud maturation point (BMP). The AXBs of group 1 are in an initial developmental stage, while those of group 2 are in a further stage of development. After short photoperiod exposure the AXBs of these groups occupied the new positions 11-15 and 16-20, respectively. Below the BMP in long photoperiod plants AXBs are para-dormant, and classified as mature (group 3) or ageing (group 4). After the short photoperiod exposure they occupied positions 21-25 and 25-30, respectively. Bud numbers are approximate.
