# Supplementary material for: Long and short photoperiod buds in hybrid aspen share structural development and expression patterns of marker genes
Source: J Exp Bot. 2015 Aug 5;66(21):6745–60. doi: 10.1093/jxb/erv380 (PMC4623686; doi:10.1093/jxb/erv380)
Supplement: Supplementary Data [file supp_erv380_Supplementary_Fig._S5._legend.pptx]

## Slide 1
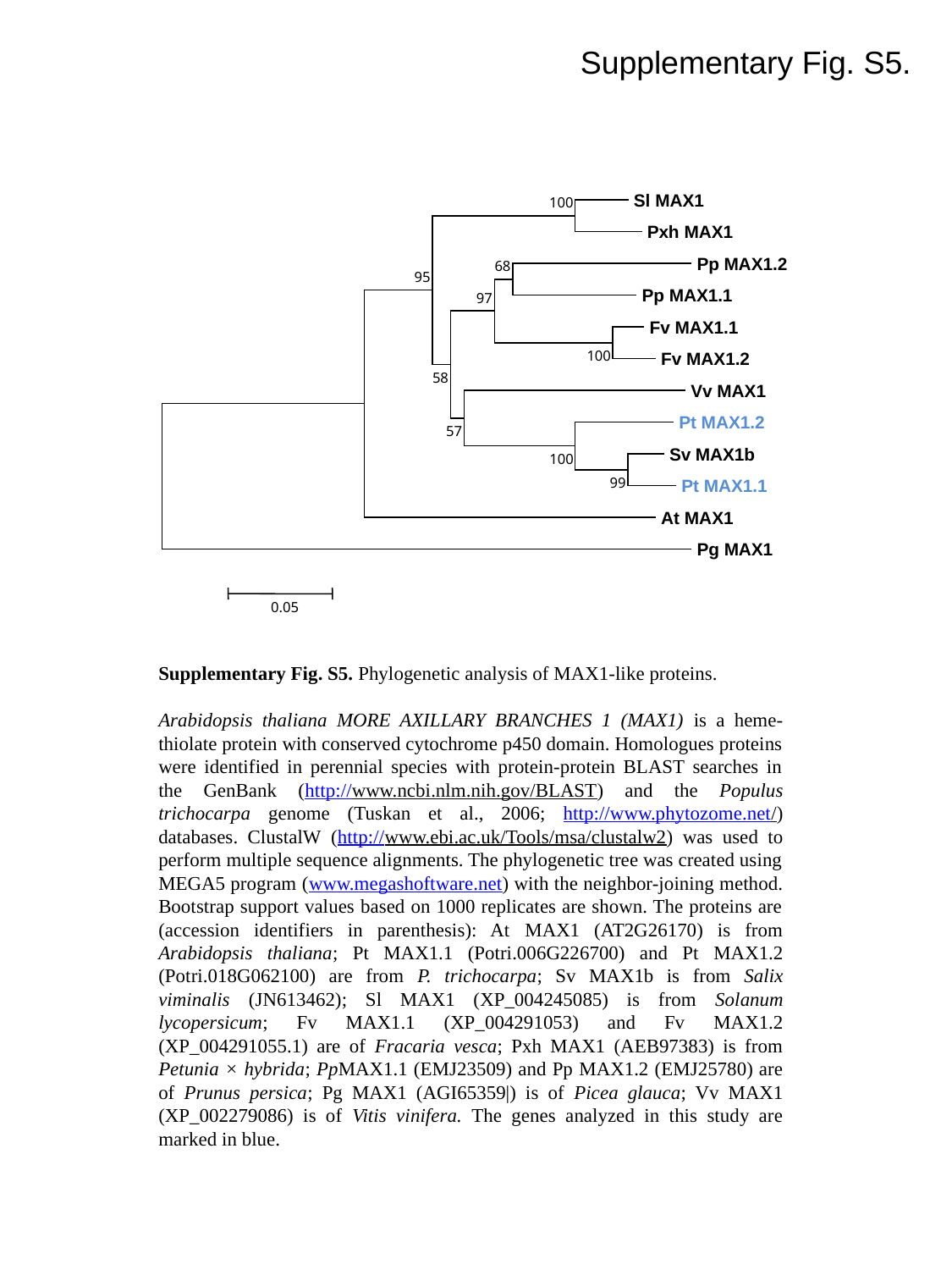

Supplementary Fig. S5.
 Sl MAX1
100
 Pxh MAX1
 Pp MAX1.2
68
95
 Pp MAX1.1
97
 Fv MAX1.1
100
 Fv MAX1.2
58
 Vv MAX1
 Pt MAX1.2
57
 Sv MAX1b
100
99
 Pt MAX1.1
 At MAX1
 Pg MAX1
0.05
Supplementary Fig. S5. Phylogenetic analysis of MAX1-like proteins.
Arabidopsis thaliana MORE AXILLARY BRANCHES 1 (MAX1) is a heme-thiolate protein with conserved cytochrome p450 domain. Homologues proteins were identified in perennial species with protein-protein BLAST searches in the GenBank (http://www.ncbi.nlm.nih.gov/BLAST) and the Populus trichocarpa genome (Tuskan et al., 2006; http://www.phytozome.net/) databases. ClustalW (http://www.ebi.ac.uk/Tools/msa/clustalw2) was used to perform multiple sequence alignments. The phylogenetic tree was created using MEGA5 program (www.megashoftware.net) with the neighbor-joining method. Bootstrap support values based on 1000 replicates are shown. The proteins are (accession identifiers in parenthesis): At MAX1 (AT2G26170) is from Arabidopsis thaliana; Pt MAX1.1 (Potri.006G226700) and Pt MAX1.2 (Potri.018G062100) are from P. trichocarpa; Sv MAX1b is from Salix viminalis (JN613462); Sl MAX1 (XP_004245085) is from Solanum lycopersicum; Fv MAX1.1 (XP_004291053) and Fv MAX1.2 (XP_004291055.1) are of Fracaria vesca; Pxh MAX1 (AEB97383) is from Petunia × hybrida; PpMAX1.1 (EMJ23509) and Pp MAX1.2 (EMJ25780) are of Prunus persica; Pg MAX1 (AGI65359|) is of Picea glauca; Vv MAX1 (XP_002279086) is of Vitis vinifera. The genes analyzed in this study are marked in blue.
