# Supplementary material for: Long and short photoperiod buds in hybrid aspen share structural development and expression patterns of marker genes
Source: J Exp Bot. 2015 Aug 5;66(21):6745–60. doi: 10.1093/jxb/erv380 (PMC4623686; doi:10.1093/jxb/erv380)
Supplement: Supplementary Data [file supp_erv380_Supplementary_Table_1.pdf]

Supplementary Table S1

Populus trichocarpa					
**Protein**	**Gene abb.**	**Locus name v2.0**	**Updated v3.0 locus name**	**Forward 5'-3'**	**Reverse 5'-3'**
Flowering time genes					
FLOWERING LOCUS T	*FT1*	POPTR_0008s07730	Potri.008G077700	GCGAGCTCAAGCCCTCTCA	TGCATCAGGGTCCACCATAAC
FLOWERING LOCUS T	*FT2*	POPTR_0010s18680	Potri.010G179700	GAGGTTGTGTGCTACGAGAGC	CACTGTTTGCCTGCCTAGTTG
CENTRORADIALIS-LIKE1	*CENL1*	POPTR_0004s21410	Potri.004G203900	AGTCCAACAGGAAGCAGGTTTTT	AAAGGATCTCATATCACCTCCATGAA
CENTRORADIALIS-LIKE2	*CENL2*	POPTR_0009s16670	Potri.009G165100	AGCTTTTCCCATCTGCTGTC	CCTGGAACATCTGGGTCTGT
CONSTANS	*CO*	POPTR_0004s10800	Potri.004G108300	GATGTTGGAGTGGTGCCAGAA	TGGATAGCAGTGCTGGAGAAAAG
BROTHER OF FT	*BFT*	POPTR_0015s13370	Potri.015G141300	ACAGACCCTGATGCTCCAAG	CAGTTGTGCCAGGAATGTCA
Genes affecting axillary shoot branching					
MORE AXILLARY BRANCHES1	*MAX1.1*	POPTR_0006s24320	Potri.006G226700	AAACGTTATGGCCCCATTTT	TGAGATGGGAGAGGGAACAC
MORE AXILLARY BRANCHES1	*MAX1.2*	POPTR_0018s07540	Potri.018G062100	CAGATGCCAACAGCTCAAGA	TCCAGGTGCTAACCAGATCC
BRANCHED1	*BRC1-LIKE*	POPTR_0012s05660	Potri.012G059900	CATCATCGCGTAAAACTCCA	GTCGATTCTTCGACTGCACA
BRANCHED2	*BRC2-LIKE*	POPTR_0010s14030	Potri.010G130200	ACTTGCTGCCACATCAAATG	AGTAGCCCCACTTGGAACCT
PINFORMED	*PIN-LIKE1*	POPTR_0012s04470	Potri.012G047200	TGTTAGGCTCGCTGTATCTCC	TGCAGCACCAACTTTCTCAC
PINFORMED	*PIN-LIKE2/PttPIN3*	POPTR_0006s03540	Potri.006G037000	CAATGACCTTGGTGGGGTAG	GCTGTGGAGCTTGAAGCAA
Meristem identity and organization					
WUSCHEL	*WUS-LIKE1*	POPTR_0005s11680	Potri.005G114700	GGTGGTGAATCCATGAACAA	AATCTTTTCAGCGGCTTCCT
WUSCHEL	*WUS-LIKE2*	POPTR_0007s14130	Potri.007G012100	ATTTCCAATGCTTGGTGAGG	ACGGGAAGAAGTGTCATTGC
CLAVATA1	*CLV1-LIKE*	POPTR_0005s26300	Potri.005G241500	TCTCGCTAAGTTCTTACAAGATGC	ACACCAAAACTGTAAACATCACTC
CLAVATA3	*CLV3-LIKE*	POPTR_0001s05380	Potri.001G016100	TGCAATGCATGAAACAACAG	CTCCCTTCCCCATTCTTGTG
KNOTTED1	*KN1-LIKE*	POPTR_0002s11400	Potri.002G113300	GCCAGGCAGAAGCTACTCAG	CCAATGTCGCTTCCTTTGAT
SHOOT MERISTEMLESS	*STM-LIKE1*	POPTR_0004s00650	Potri.004G004700	ACATGTTAATGCGGGGGATT	TGTTGTTGTGGTTGTGATCG
SHOOT MERISTEMLESS	*STM-LIKE2*	POPTR_0011s01600	Potri.011G011100	GTCTCCTTGCTGCCTATGCT	GCAATAGAAGCAGCCGATG
Reference gene actin					
ACTIN	*ACT*	POPTR_0001s31700	Potri.001G309500	CGATGCCGAGGATATTCAAC	ACCAGTGTGTCTTGGTCTACCC
