# Supplementary material for: Long and short photoperiod buds in hybrid aspen share structural development and expression patterns of marker genes
Source: J Exp Bot. 2015 Aug 5;66(21):6745–60. doi: 10.1093/jxb/erv380 (PMC4623686; doi:10.1093/jxb/erv380)
Supplement: Supplementary Data [file supp_erv380_Supplementary_Fig._S4._legend.pptx]

## Slide 1
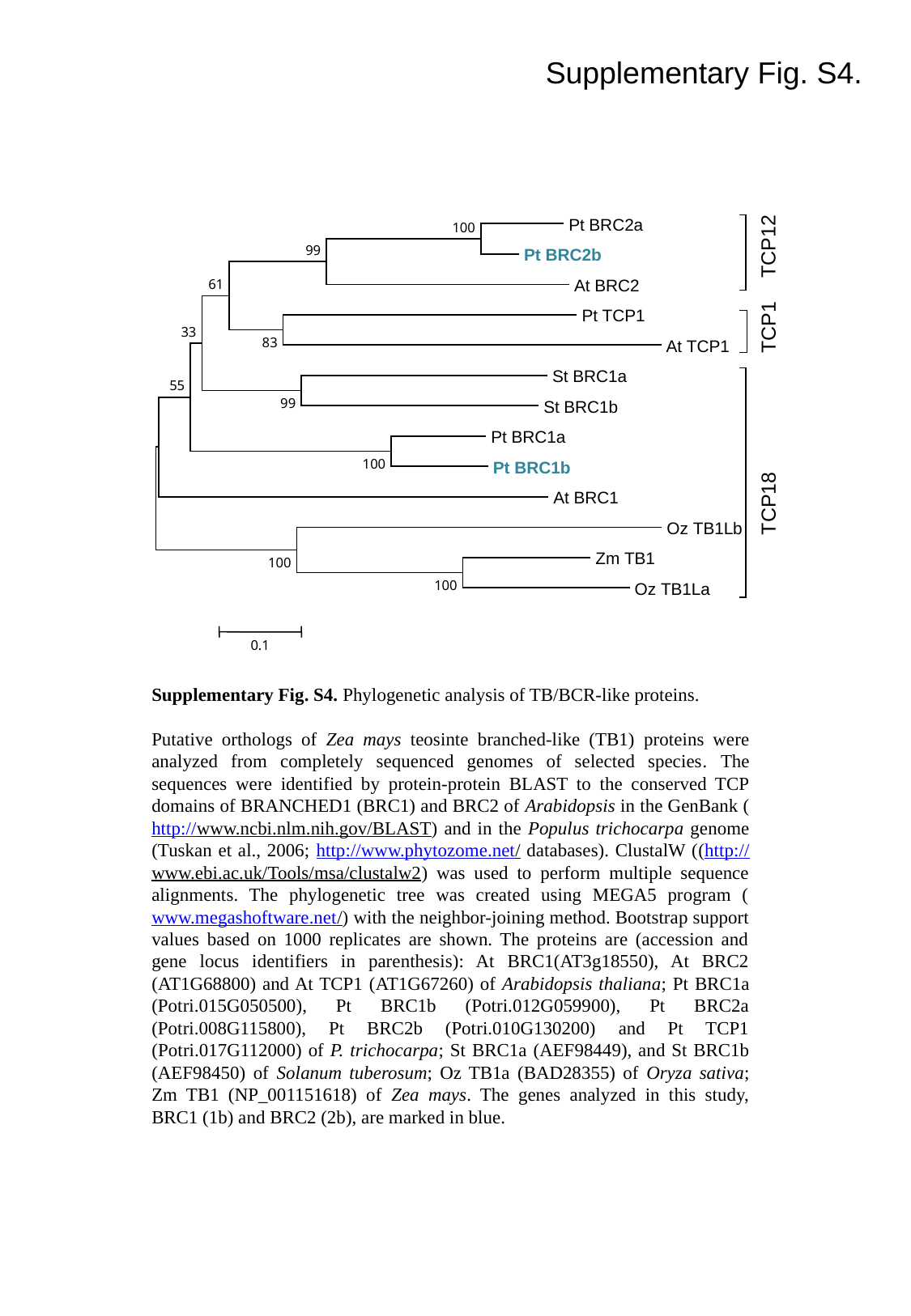

Supplementary Fig. S4.
 Pt BRC2a
100
99
 Pt BRC2b
 At BRC2
61
 Pt TCP1
33
83
 At TCP1
 St BRC1a
55
99
 St BRC1b
 Pt BRC1a
100
 Pt BRC1b
 At BRC1
 Oz TB1Lb
 Zm TB1
100
100
 Oz TB1La
0.1
TCP12
TCP1
TCP18
Supplementary Fig. S4. Phylogenetic analysis of TB/BCR-like proteins.
Putative orthologs of Zea mays teosinte branched-like (TB1) proteins were analyzed from completely sequenced genomes of selected species. The sequences were identified by protein-protein BLAST to the conserved TCP domains of BRANCHED1 (BRC1) and BRC2 of Arabidopsis in the GenBank (http://www.ncbi.nlm.nih.gov/BLAST) and in the Populus trichocarpa genome (Tuskan et al., 2006; http://www.phytozome.net/ databases). ClustalW ((http://www.ebi.ac.uk/Tools/msa/clustalw2) was used to perform multiple sequence alignments. The phylogenetic tree was created using MEGA5 program (www.megashoftware.net/) with the neighbor-joining method. Bootstrap support values based on 1000 replicates are shown. The proteins are (accession and gene locus identifiers in parenthesis): At BRC1(AT3g18550), At BRC2 (AT1G68800) and At TCP1 (AT1G67260) of Arabidopsis thaliana; Pt BRC1a (Potri.015G050500), Pt BRC1b (Potri.012G059900), Pt BRC2a (Potri.008G115800), Pt BRC2b (Potri.010G130200) and Pt TCP1 (Potri.017G112000) of P. trichocarpa; St BRC1a (AEF98449), and St BRC1b (AEF98450) of Solanum tuberosum; Oz TB1a (BAD28355) of Oryza sativa; Zm TB1 (NP_001151618) of Zea mays. The genes analyzed in this study, BRC1 (1b) and BRC2 (2b), are marked in blue.
